# Serum exosomal proteomics analysis of lung adenocarcinoma to discover new tumor markers

**DOI:** 10.1186/s12885-022-09366-x

**Published:** 2022-03-15

**Authors:** Shanshan Liu, Wenjuan Tian, Yuefeng Ma, Jiaji Li, Jun Yang, Burong Li

**Affiliations:** 1 Department of Clinical Laboratory, The Second Affiliated Hospital of Xi’an Jiaotong University, Xi’an, Shaanxi 710004 P. R. China; 2 Department of Clinical Laboratory, The First Affiliated Hospital of Xi’an Jiaotong University, Xi’an, Shaanxi 710004 P. R. China; 3 Internal Medicine Laboratory, Second Hospital of Shanxi Medical University, Taiyuan, Shanxi 030001 P. R. China; 4 Department of Thoracic Surgery, The Second Affiliated Hospital of Xi’an Jiaotong University, Xi’an, Shaanxi 710004 P. R. China; 5 The Second Clinical Medical College, Shaanxi University of Traditional Chinese Medicine, Xianyang, Shaanxi 712046 P. R. China; 6 Department of Pathology, The Second Affiliated Hospital of Xi’an Jiaotong University, Xi’an, Shaanxi 710004 P. R. China

**Keywords:** Serum exosomes, Proteomics, Tumor markers, Lung adenocarcinoma

## Abstract

**Background:**

Among the most aggressive and rapidly lethal types of lung cancer, lung adenocarcinoma is the most common type. Exosomes, as a hot area, play an influential role in cancer. By using proteomics analysis, we aimed to identify potential markers of lung adenocarcinoma in serum.

**Methods:**

In our study, we used the ultracentrifugation method to isolate serum exosomes. The Liquid chromatography-mass spectrometry (LC–MS) and bioinformatics analysis were used to identify potential serum exosomal proteins with altered expression among patients with advanced lung adenocarcinoma, early lung adenocarcinoma, and healthy controls. A western blot (WB) was performed to confirm the above differential expression levels in a separate serum sample-isolated exosome, and immunohistochemistry (IHC) staining was conducted to detect expression levels of the above differential proteins of serum exosomes in lung adenocarcinoma tissues and adjacent tissues. Furthermore, we compared different expression models of the above differential proteins in serum and exosomes.

**Result:**

According to the ITGAM (Integrin alpha M chain) and CLU (Clusterin) were differentially expressed in serum exosomes among different groups as well as tumor tissues and adjacent tissues. ITGAM was significantly and specifically enriched in exosomes. As compared to serum, CLU did not appear to be significantly enriched in exosomes. ITGAM and CLU were identified as serum exosomal protein markers of lung adenocarcinoma.

**Conclusions:**

This study can provide novel ideas and a research basis for targeting lung adenocarcinoma treatment as a preliminary study.

**Supplementary Information:**

The online version contains supplementary material available at 10.1186/s12885-022-09366-x.

## Background

Lung cancer is the leading cause of cancer deaths among men and the second leading cause of cancer deaths among women globally, accounting for about 1/5 of all cancer deaths worldwide, according to the latest edition of the global cancer statistics [1]. Among smokers and non-smokers, adenocarcinoma of the lung is the most common type [2, 3]. Moreover, lung adenocarcinoma is one of the most aggressive and rapidly lethal cancers, with a median survival rate of less than 5 years [4]. It is therefore expected that the mortality of lung cancer will be reduced with an in-depth understanding of how lung adenocarcinomas develop, the discovery of novel tumor markers, as well as the development of new drugs and therapeutic modalities.

It is not only the genomic changes and molecular characteristics of cancer cells that initiate and progress lung cancer however, their interaction with the tumor microenvironment, particularly the immune system, is critical [5]. On the one hand, immunological surveillance suppresses tumor growth while on the other hand, tumor cells alter the function of the immune system through immunological editing. As a result, the immune system not only reduces the killing of tumor cells but also promotes the proliferation of tumor cells [6]. The tumor microenvironment includes different cells (endothelial cells, fibroblasts, immune cells, etc.), extracellular components (cytokines, growth factors, hormones, extracellular matrix, etc.) as well as the vascular and nervous system [7].

Exosomes belong to extracellular vesicles (EVs) which mainly include three types: exosomes, microvesicles, and apoptotic bodies [8]. Exosomes, essentially intraluminal vesicles (ILVs), are formed by the process that the inward budding of early endosomes produces multivesicular endosomes or multivesicular bodies (MVBs) and their fusion with cell membranes followed by exosomes release. Besides, MVBs may be degraded by fusing with lysosomes and the fate of MVBs depends on the function states of cells [9, 10].

Numerous pieces of researches have shown that exosomes, as the “functional agents” of cells, carry proteins with important functions, and act as transport mechanisms for them to reach their targets through binding to receptors, fusion with membranes, and internalization of vesicles. Therefore, through the transfer of active molecules, exosomes participate in communication within tumor cells, as well as between tumor cells and tumor microenvironment cells [8, 11]. Exosomes are capable of interacting with recipient cells in the local environment by paracrine action, or in distant tissues through endocrine action. Exosomes present in the circulatory system are mainly released from three sources: circulating blood cells (platelets, lymphocytes, dendritic cells, and other immune cells), vascular wall cells (such as endothelial cells), tumor tissue in cancer patients [12–14]. A large number of exosomal proteins in the circulatory system are related to immune responses. They may be released by the immune cells in the tumor microenvironment, which are edited by cancer cells-derived exosomes in tumor tissues, or they may come from immune cells of the circulatory system. In short, these exosomal proteins in the circulatory system can reflect the body’s immune landscape in normal and pathological states [14, 15], meanwhile, the immune microenvironment is related to tumor progression in metastatic organs [16]. Besides, serum samples are readily available in clinical settings and exosomes are stable in blood and other biological fluids playing a role in their ability to target specific tissues at a long-distance [17]. Therefore, serum exosomal proteins can serve as potential tumor markers, which can reflect the body’s immune status, disease staging and treatment response [15, 18].

In summary, our study aimed to identify possible serum exosomal markers of lung adenocarcinoma using liquid chromatography-mass spectrometry (LC-MS) technology and Western blot (WB). ITGAM and CLU were identified as serum exosomal protein markers of lung adenocarcinoma.

## Materials and methods

### Source and grouping of specimens

Serum samples were collected from the blood of lung adenocarcinoma patients and healthy subjects in the clinical laboratory of the Second Affiliated Hospital of Xi’an Jiaotong University from July 2018 to January 2019. We defined early lung adenocarcinoma patients as those with cancer stages I-IIIA, and advanced lung adenocarcinoma patients as those with cancer stages IIIB-IV in our study. A total of 39 serum specimens were analyzed. We conducted proteomics analysis of serum exosomes by LC-MS using 9 samples which were three each in the advanced lung adenocarcinoma group, early lung adenocarcinoma group, and healthy control, group. Another 27 samples of three groups were used for WB to validate these potentially differential proteins of exosomes. The remaining 3 samples of the advanced lung adenocarcinoma group were used to make comparisons of different expression models of ITGAM and CLU in exosomes and serum. The clinical information of individuals providing serum samples was shown in Supplementary Table 1 (①, ②, and ③ referred to the WB grouping). There were three patients in each group of WB (advanced lung adenocarcinoma, early lung adenocarcinoma, and healthy control).

Archived tissue paraffin blocks were collected from the department of pathology, the Second Affiliated Hospital of Xi’an Jiaotong University from January 2019 to June 2019, including 15 lung adenocarcinoma tissue blocks and 5 adjacent tissue blocks. Clinical data of these patients were shown in Supplementary Table 2.

### Exosomes isolation

For ultracentrifugation to isolate exosomes, serum was pre-purified by a series of centrifugation at 300 x g for 5 min, 2,000 x g for 10 min, and 10,000 x g for 30 min at 4 ℃. Then the supernatant was diluted using sterile 1×PBS at 1:4. The diluted supernatant was subsequently centrifuged at 11,0000g for 75 min at 4 ℃ (Beckman Optima 100-XP ultracentrifuge, USA), resuspended in PBS, and filtered with a 0.22um filter. Repeat the above steps.

### Exosomal protein extraction and bicinchoninic acid (BCA) protein assay

Each exosome sample was mixed with an equal volume of protein lysis buffer. The mixture was then incubated, vortexed, and centrifuged. The protein concentration was determined by BCA protein assay (Boster Biological Technology Co., Ltd. Wuhan of China).

### Exosome characterization

#### Nanoparticle tracking analysis (NTA)

Exosome size and particle number were analyzed using nanoparticle characterization system (ZetaVIEW S/N 17-310, PARTICLE METRIX, German) and ZetaView 8.04.02 software.

#### Transmission electron microscopy (TEM)

We used TEM (Hitachi H-7650, Japan) to assess the size and morphology of exosomes. Around 10 µL of the exosome suspension was deposited on copper grids and treated with 2% uranyl acetate for 10 min at room temperature. The sample was visualized using an electron microscope.

#### Western blotting analysis of exosomal markers

Exosomal proteins were separated by 10% sodium dodecyl sulfate-polyacrylamide gel electrophoresis (SDS–PAGE kits, Boster Biological Technology Co., Ltd. Wuhan of China) and transferred onto 0.22 μm polyvinylidene fluoride (PVDF) membranes (Millipore, USA) by wet electro-transfer. Membranes were blocked in 5% non-fatty milk for 1 h. Then primary antibodies were added for overnight incubation at 4 ℃ (anti-CD9: SBI, EXOAB-CD9A-1, 1:1000; anti-CD81: Immunoway, YT5394, 1:1000). After washing by 0.1% PBST (10 min × 3 times), the membranes were incubated with the horseradish peroxidase (HRP)-conjugated secondary antibody (Elabscience, E-AB-1003, 1:4000) for 1 h at room temperature. After washing by 0.1% PBST (10 min × 3 times), enhanced chemiluminescence (ECL) detection kit (Boster Biological Technology Co., Ltd. Wuhan of China) was used to detect protein bands by Tanon-5200 automatic chemiluminescence imaging analysis system (Tanon Science & Technology Co., Ltd. Shanghai of China).

### LC–MS analysis of exosomal proteins

Firstly we prepared the samples for LC-MS analysis. The exosomal proteins were mixed with acetone overnight at -20°C. Centrifuged pellets were washed in pre-cooled ethanol, acetone, and acetic acid, and centrifuged again. Pellets were re-dissolved with guanidine hydrochloride and TEAB. The protein was then mixed with NH4HCO3 and DTT and incubated for 1 h. Then 10 μL of 1 M iodoacetamide was added to the above mixture and incubated for 40 min at room temperature in the dark. A Ziptip C18 column was used for concentration washing, trypsinization, and desalting (Millipore).

Secondly, we used LC-MS for qualitative and quantitative analysis of proteins. On-line Nano-Reversed Phase Liquid Chromatography (RPLC) was performed on Easy-nLC 1000 system (Thermo Scientific); The analysis column was a C18 reversed-phase chromatography column (PepMap100, C18, 2 μm, 50 μm × 150 mm NanoViper, Thermofisher Dionex), and the gradient used in the experiment was to increase the mobile phase B from 2% to 40% within 103 minutes; Q Exactive plus system (Thermo Scientific) was used for Mass spectrometer analysis with nanoliter spray ESI ion source, 1.6 kV of spray voltage and 275 °C of capillary temperature. The spectrometer was operated in data-dependent mode with survey scans acquired at a resolution of 70,000 in MS mode, and 17,500 in MS/MS mode. A series of optimizations led to the use of MS/MS data containing fragment ion information for relative protein quantification, and MS/MS data containing peptide peak intensity for protein identification using Swissprot human databases with MaxQuant software (MaxQuant 1.5.8.3, Max-Planck Institute for Biochemistry, Germany) after a series of similarity comparisons. The peptide spectrum matches (PSM) FDR was less than 0.01, as well as the protein FDR 0.05 and the site FDR 0.01.

### Bioinformatics analysis

We used Gene Set Enrichment Analysis (GSEA) software (version: 4.0.3) to conduct functional enrichment analysis of exosomal proteins (These gene sets with statistical significance met criteria of nominal *p*-value < 0.05, FDR *q*-value < 0.25 and NES > 1). The raw data for GSEA could be seen in Supplementary Table 3. Therefore, we obtained relatively higher expressed differential protein lists, which were determined by score value based on Signal2Noise from GSEA. Then, we made an intersection of the above protein lists. String database (https://string-db.org/) was used to analyze functions of the protein intersection. The expression levels of these proteins in lung adenocarcinoma tissues and adjacent tissues were further studied using Metabolic Gene RApid Visualize (http://merav.wi.mit.edu/) and Kaplan Meier plotter (https://kmplot.com/analysis/) databases.

### WB analysis of differentially expressed exosomal proteins

The detailed process of WB has been explained above in part 2.4.3, and the difference is that we cut the gel before antibody hybridization. Information on primary antibodies was as follows: ITGAM, Abcam, ab133357, 1:1000; CLU, Proteintech, 12289-1-AP, 1:500; Alix, Proteintech, 12422-1-AP, 1:1000. Alix, as the exosomal marker, was considered as a loading control [19].

### IHC staining

Tissue sections were deparaffinized in 2 xylene baths for 15 minutes, followed by a rehydration process in which sections were incubated in 100%, 95%, and 50% ethanol for 2 minutes in each bath. Slices are hydrated. Treat with 0.1% Triton X-100 for 15 minutes. We performed the staining procedure using the SABC-POD kit (Wuhan Bost Biotechnology Co., Ltd., China). Primary antibody (ITGAM, Abcam, ab133357, 1:4000; CLU, Proteintech, 12289-1-AP, 1:50) was incubated overnight at 4 °C. DAB kit (Wuhan Bost Biotechnology Co., Ltd., China) was used for section color development. We used a digital pathology scanner (NanoZoomer 2.0-RS, Japan) to obtain staining information for the whole section. Image-Pro Plus software was used to obtain the integrated option density (IOD) of each selected image, and the average IOD of all selected images in the whole section represented the protein expression level of the whole section.

### Statistical analysis

Data were analyzed using SPSS 22.0 and Graphpad Prism 7.0. For the analysis of WB results, the comparison among different groups (advanced lung adenocarcinoma group, early lung adenocarcinoma, and healthy control group) was performed by one-way analysis of variance (ANOVA), and Bonferroni test was used for afterward multiple-comparison of three means. For the analysis of IHC results, an unpaired t-test was used to analyze the difference between two groups (lung adenocarcinoma tissue group and adjacent tissue group). Less than 0.05 of the *p*-value was considered as a statistically significant difference. For the Bonferroni test, we considered differential expression between two groups as *p* < 0.05/3 = 0.0167.

## Results

### Isolation and characterization of serum exosomes

**Fig. 1:**
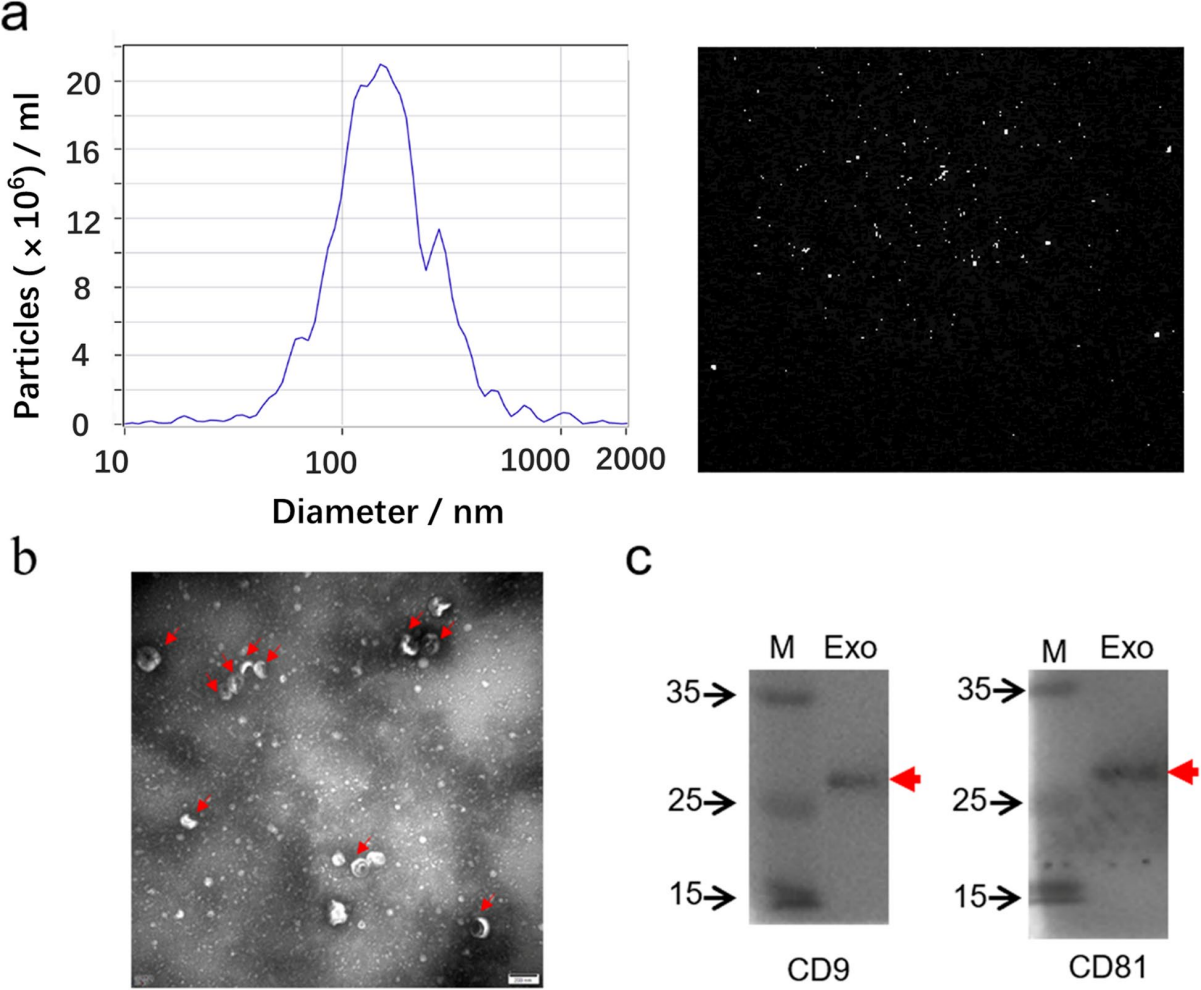
Characterization of serum exosomes. **a** NTA showed that the average size of the exosome population was 145.8 nm. **b** The view of TEM displayed the cup-shaped morphology of exosomes. **c** WB results displayed that CD9 and CD81, as exosomal markers, were expressed in isolated exosomes

We used ultracentrifugation isolating serum exosomes. According to NTA, the average size of the exosome population was 145.8 nm, which was consistent with typical sizes of exosomes within 30-150 nm (Fig. 1a). The TEM image displayed the cup-shaped morphology of exosomes (Fig. 1b). Moreover, WB results displayed that CD9 and CD81, as exosomal markers, were expressed in isolated exosomes (Fig.1c).

### Results of LC–MS analysis combined bioinformatic analysis

As part of the study, LC-MS was used to both qualitatively and quantitatively evaluate differences between early lung adenocarcinoma and advanced lung adenocarcinoma groups, as well as healthy controls. There was a total of 627 proteins identified in the nine exosome samples. We evaluated the results of protein identification in three aspects including the number of unique peptides, peptides length, and protein coverage in Supplementary Figure 1. In general, proteins with high reliability were considered to contain ≥ 2 unique peptides. Supplementary Fig. 1a displayed that the number of proteins containing ≥ 2 unique peptides in this study was 495, accounting for 78.95% (495/627) of the total protein number. Peptides are too long or too short to be detected in a mass spectrometer. Generally, too short or too long length of peptides reflects the inappropriate selection of the protease. Supplementary Fig. 1b showed that the maximum peptide length was 9, and the average length was 14.47, which was in line with the reasonable range of peptide length. Protein coverage refers to the ratio of the number of amino acids in the identified peptides to the total number of amino acids in the protein sequence, which reflects the overall accuracy of protein identification results. Supplementary Fig. 1c displayed that proteins with coverage ≥20% accounted for 54.42% of the total proteins, and the average coverage of proteins was 26.28%.

**Fig. 2:**
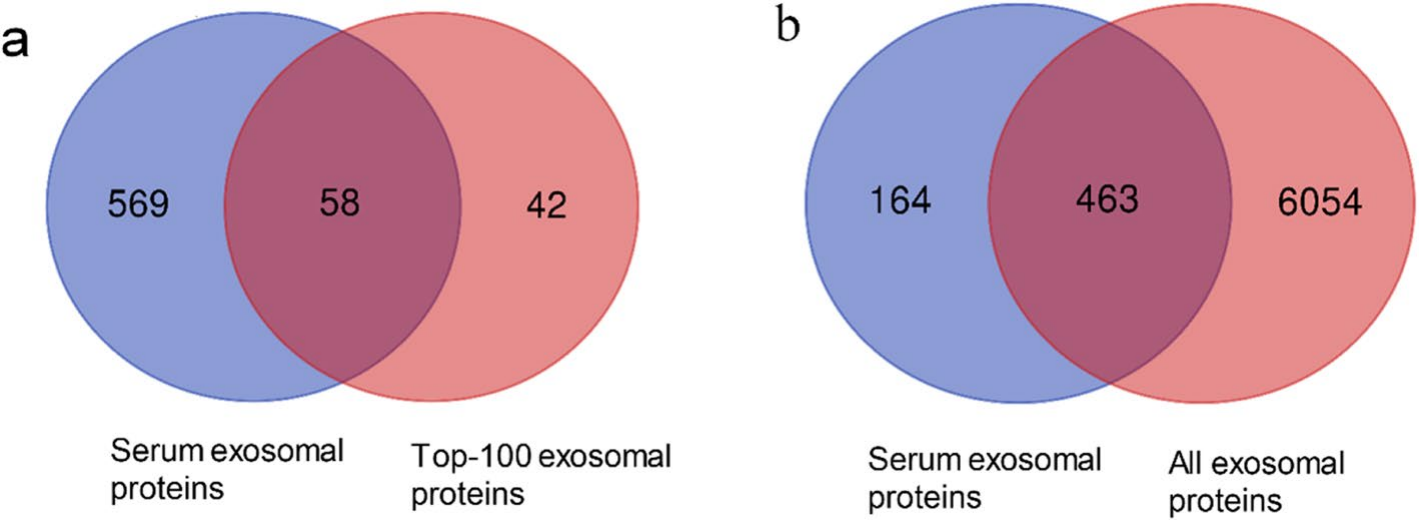
Venn diagrams of serum exosomal proteins against the Exocarta database. **a** The protein intersection between our results and the protein list which included the most identified 100 exosomal proteins from the Exocarta database. **b** The protein intersection between our results and the protein list which contained all exosomal proteins from the Exocarta database

Exocarta, an exosome database, provides the information of exosomal content from reported literature. The Exocarta database contains the top 100 identified exosomal proteins, and our results contain 58 common proteins (www.exocarta.org) (Fig. 2a). Moreover, there was a total of 463 shared proteins between our results and the protein list which contained all exosomal proteins (6517 proteins) from the Exocarta database (Fig. 2b).

**Fig. 3:**
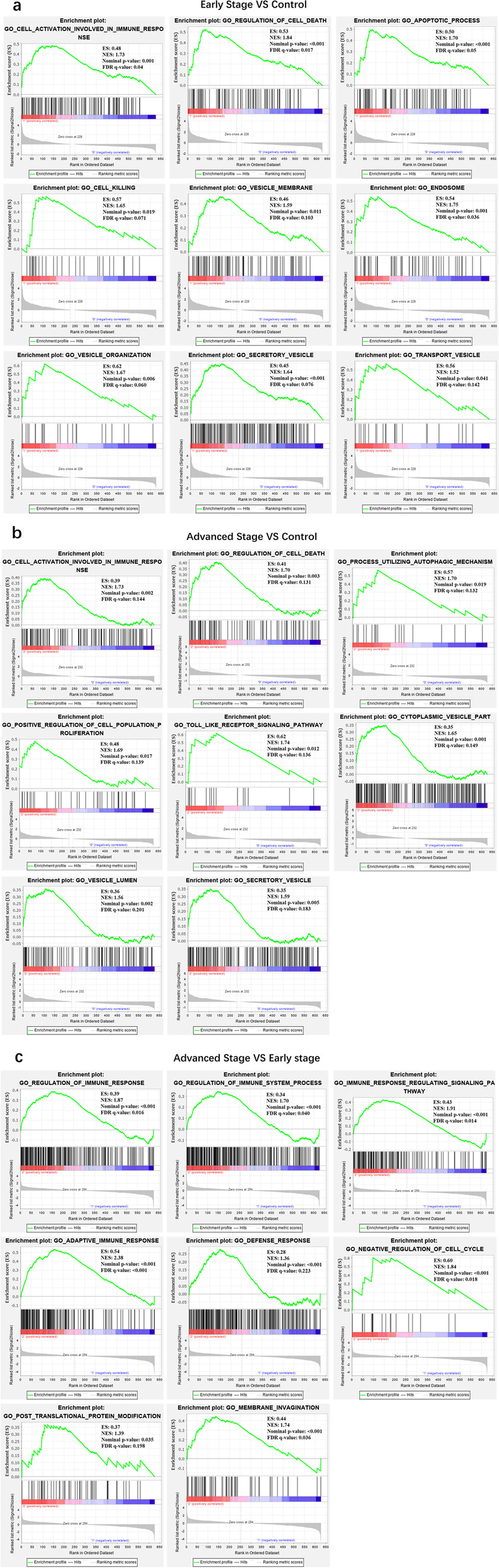
GSEA analysis. **a** These gene sets were relatively up-regulated in the early lung adenocarcinoma group compared with the healthy control group. **b** These gene sets were relatively up-regulated in the advanced lung adenocarcinoma group compared with the healthy control group. **c** These gene sets were relatively up-regulated in the advanced lung adenocarcinoma group compared with the early lung adenocarcinoma group

**Fig. 4:**
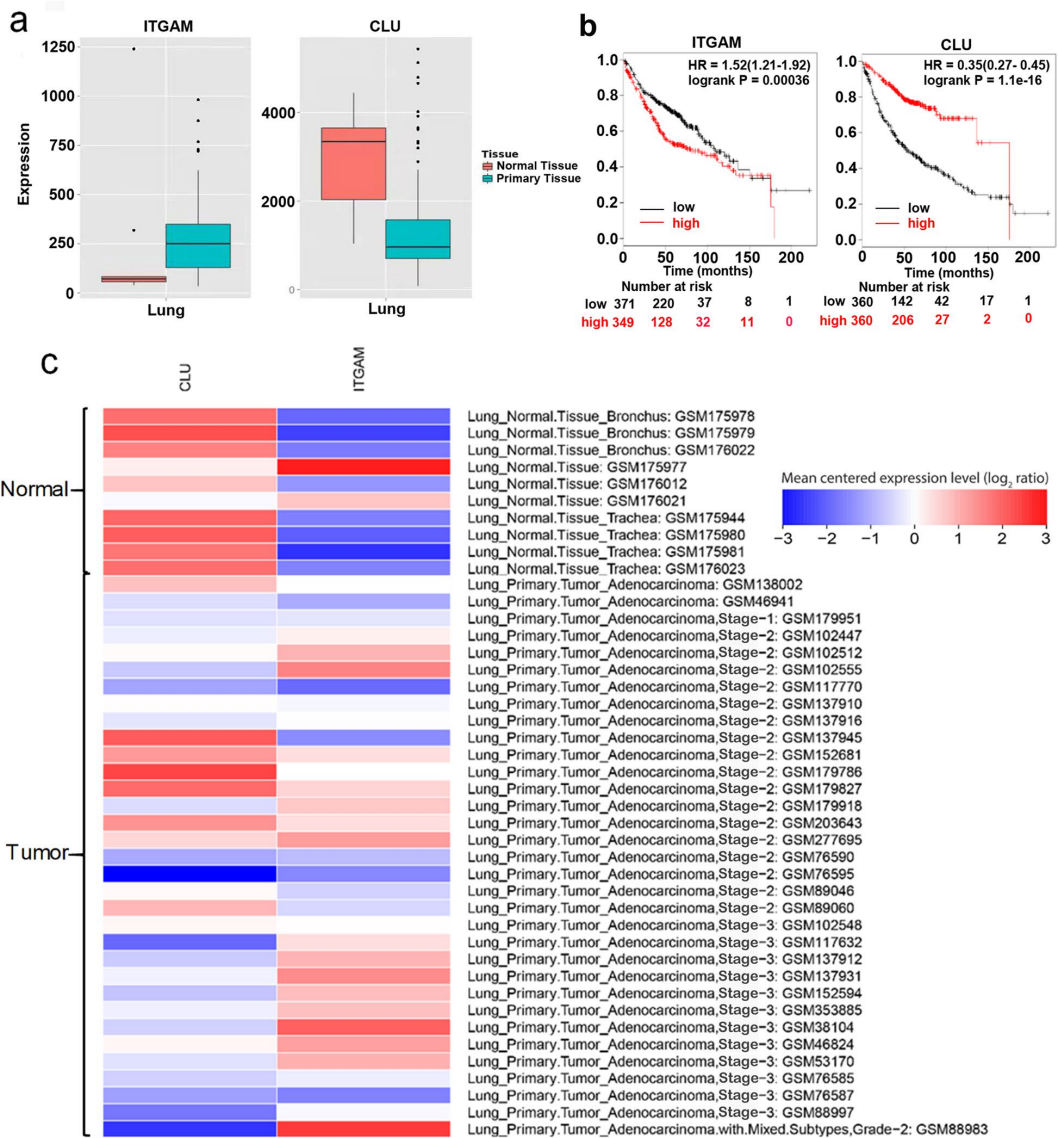
Bioinformatics analysis in websites of Metabolic gEne RApid Visualize and Kaplan Meier plotter. **a** Boxplot showed expression levels of ITGAM and CLU in lung adenocarcinoma tissues and adjacent tissues. The horizontal line represented the median expression level of proteins. The top and bottom of the vertical line represented the upper quartile (75%) and lower quartile (25%). These dots in the box plot are representing samples whose protein expression levels were more than the upper quartile (75%). **b** The survival curve of ITGAM showed that the expression level of ITGAM in lung tissues was related to a poor prognosis, while CLU with a favorable prognosis. **c** Heatmap displayed expression levels of ITGAM and CLU in lung adenocarcinoma tissues and adjacent tissues

We used GSEA to analyze the function of these serum exosomal proteins. During the analysis of groups that differed in stage, we focused on these gene sets that were relatively up-regulated in the higher stage group. For example, when comparing the early adenocarcinoma group to the healthy control group we focused more on these gene sets that were relatively up-regulated. Figure 3a displayed these gene sets which were relatively up-regulated in the early lung adenocarcinoma group compared with the healthy control group. Figure 3b showed these gene sets which were relatively up-regulated in the advanced lung adenocarcinoma group compared with the healthy control group. Figure 3c showed these gene sets which were relatively up-regulated in the advanced lung adenocarcinoma group compared with the early lung adenocarcinoma group. We found that enriched function of these gene sets could be mainly divided into three categories containing vesicle-associated pathways (Vesicle membrane, Endosome, Vesicle organization, Secretory vesicle, Transport vesicle, Cytoplastic vesicle part, Vesicle lumen, and Membrane invagination), cancer-associated pathways (Regulation of cell death, Apoptotic process, Cell killing, Process utilizing autophagic mechanism, Positive regulation of cell death, Toll-like receptor signaling pathway, and Negative regulation of cell cycle) and immune response-associated pathways. These vesicle-associated pathways were closely related to exosome biogenesis [20]. In the part of instruction, we also mentioned that these enriched pathways also showed that circulating exosomes were indeed associated with cancer immunity. Our next analysis consisted of obtaining relatively higher expressed differential protein lists between every two groups, and combining them. Then 62 common proteins were further analyzed in the String database. These proteins were significantly enriched in three pathways (Leukocyte mediated immunity, Immune effector process, and Regulation of inflammatory response). Common proteins among the above three pathways were further analyzed in websites of Metabolic gEne RApid Visualize and Kaplan Meier plotter. Finally, we considered further investigation of both the integrin alpha M chain (ITGAM) and Clusterin (CLU). Figure 4a and c showed that expression levels of ITGAM and CLU in lung adenocarcinoma tissues and adjacent tissues in the form of the boxplot and heatmap, which were obtained by the analysis in the database of Metabolic gEne RApid Visualize. ITGAM was higher expressed in tumor tissues compared with normal tissues, while CLU was higher expressed in normal tissues. Figure 4b from the Kaplan Meier plotter website displayed that the expression levels of ITGAM in lung tissues were related to a poor prognosis (HR = 1.52, *p* = 0.00036) while CLU with a favorable prognosis (HR = 0.35, *p* = 1.1e-16).

### WB analysis for validating the differential expression of serum exosomal ITGAM and CLU

**Fig. 5:**
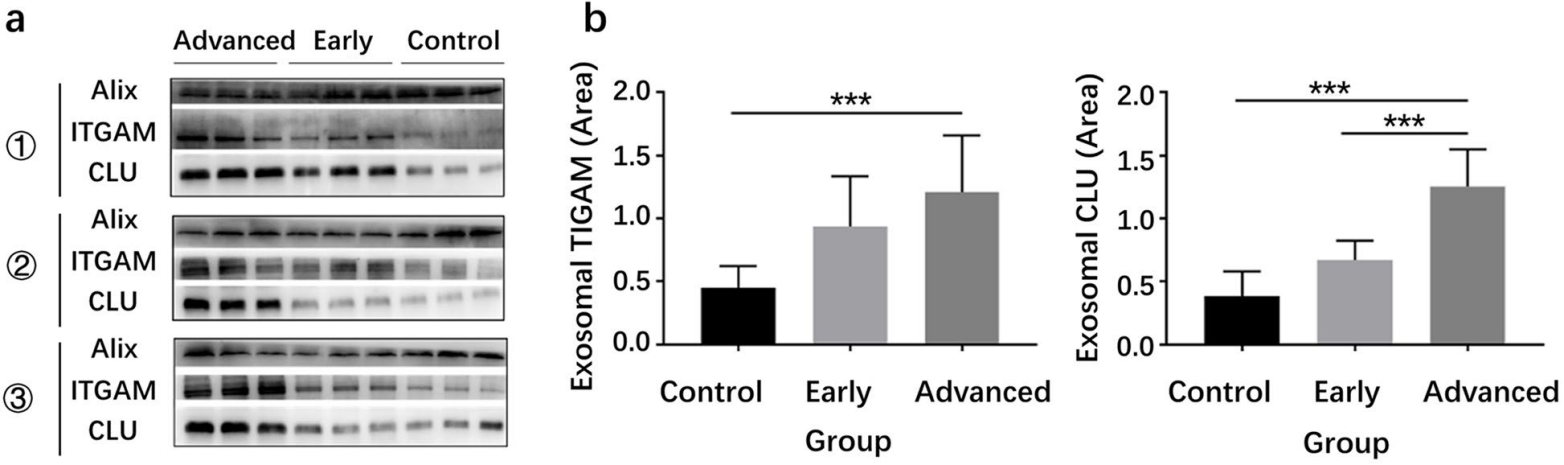
WB analysis for validating the differential expression of serum exosomal ITGAM and CLU. **a** The images of WB results of ITGAM and CLU among different groups. **b** Statistical analysis of WB results. * represented *p* < 0.05, ** represented *p* < 0.01, *** represented *p* < 0.001

We used WB to validate the differential expression of serum exosomal ITGAM and CLU among the advanced lung adenocarcinoma group, early lung adenocarcinoma group, and healthy control group. Figure 5a displayed the images of WB results. The statistical analysis indicated that ITGAM and CLU were differentially expressed with statistical significance among different groups (ITGAM, *p* = 0.001, CLU, *p* < 0.001). Figure 5b showed that serum exosomal ITGAM was higher expressed in the advanced lung adenocarcinoma group compared with the healthy control group (*p* < 0.001), and CLU was higher expressed in the advanced lung adenocarcinoma group compared with the early lung adenocarcinoma group (*p* < 0.001) and the healthy control group (*p* < 0.001).

### IHC analysis for expression levels of ITGAM and CLU in lung adenocarcinoma tissues and adjacent tissues

**Fig. 6:**
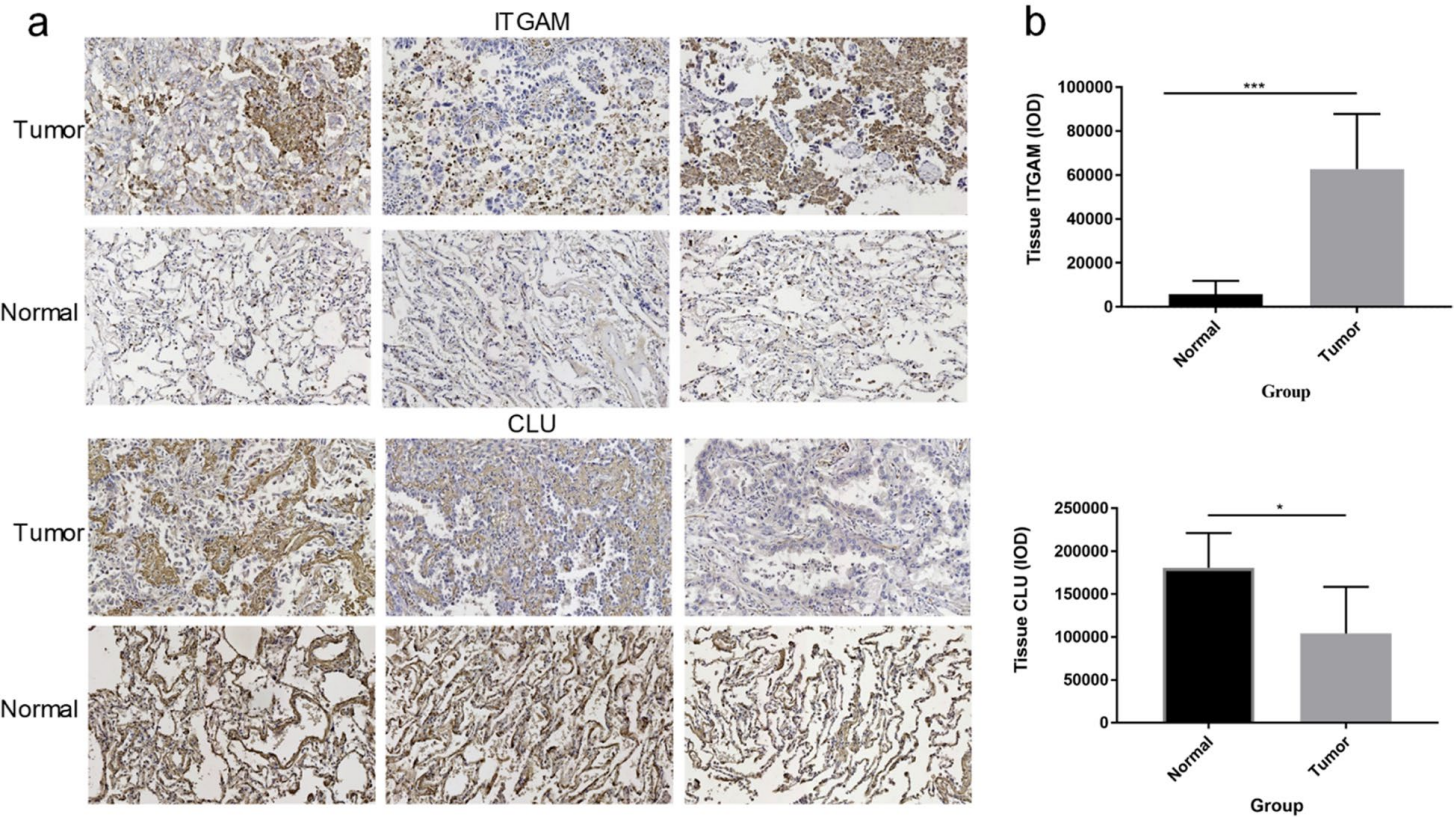
IHC analysis for expression levels of ITGAM and CLU in lung adenocarcinoma tissues and adjacent tissues. **a** Representative view of IHC staining for ITGAM and CLU. **b** Statistical analysis of IHC results. * represented *p* < 0.05, ** represented *p* < 0.01, *** represented *p* < 0.001

The IHC staining of lung adenocarcinoma tissues and nearby tissues was used to detect the expression of ITGAM and CLU. Figure 6a showed these representative views of IHC staining. For ITGAM, we found that there were weak positive and negative expressions in the adjacent tissues while strong positive and positive expressions in the tumor tissues. For CLU, strong positive and positive expressions were found in both adjacent tissues and tumor tissues, and one sample of tumor tissues showed weak positive and negative expressions. Figure 6b displayed that ITGAM had a higher expression level in tumor tissues compared with adjacent tissues (ITGAM, *p* = 0.004), while CLU with a higher expression level in adjacent tissues (CLU, *p* = 0.0117).

### The comparison of different expression models of ITGAM and CLU in exosomes and serum

**Fig. 7:**
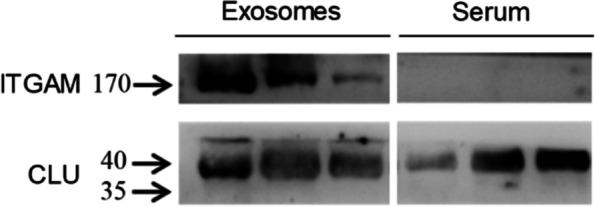
Different expression levels of ITGAM and CLU in exosomes and serum samples

We isolated serum exosomes from three advanced lung adenocarcinomas. In comparison with the expression level in serum, ITGAM was significantly and specifically enriched in exosomes. The CLU expression in serum did not appear to be significantly enriched in exosomes (Fig. 7).

## Discussion

The LC-MS method was used to identify 627 proteins from nine serum exosome samples from three patients with advanced lung adenocarcinoma, three patients with early lung adenocarcinoma, and three healthy subjects. We compare our results with those in the Exocarta database. It was found that 74% (463/627) of the exosomal proteins identified in this study appeared in the Exocarta database, and 58 proteins from the top-100 exosomal proteins from the database were identified in this study, which confirmed the effectiveness of ultracentrifugation and LC-MS in our study. We considered serum exosomal proteins ITGAM and CLU as markers for lung adenocarcinoma.

ITGAM belongs to the integrin family. In this study, the expression of serum exosomal ITGAM in the advanced lung adenocarcinoma group was significantly higher than that in the healthy control group. Compared with the expression level in serum, ITGAM was significantly and specifically enriched in exosomes. Besides, ITGAM expression in lung adenocarcinoma tissues was higher than that in adjacent tissues. Despite our literature search, we found that no studies have explored ITGAM’s potential role in lung cancer. Boguslawska et al. [21] have shown that the expression disorder of ITGAM involved in adhesion and extracellular matrix remodeling was associated with poor prognosis in patients with renal cell carcinoma. Exosome-associated studies have indicated that exosomes containing integrin facilitated the transmission of long-range adhesion signals, which determined the specifically targeted organ metastasis and promoted the formation of a premetastatic niche by the interaction of integrin with specific cells and extracellular matrix of the targeted organ [22–24]. Some studies also showed that ITGAM was related to the function of immune cells. Single nucleotide polymorphism (SNP) variations of ITGAM influenced the function of dendritic cells to present antigens and activate T cells [25], and the expression level of ITGAM was associated with the killing effect of natural killer (NK) cells [26]. Numerous studies have indicated that myeloid-derived suppressor cells (MDSCs) with high expression of ITGAM are important in promoting tumor progression [27–29]. ITGAM was also linked to implantation metastasis of epithelial ovarian cancer and liver metastasis of primary colorectal cancer, according to other research [30, 31]. As a result of the above important role of ITGAM in these reported studies, more investigation is required into the role of serum exosomal ITGAM in lung adenocarcinoma.

CLU is a heterodimeric glycoprotein linked by disulfide bonds and plays a role in tumorigenesis and progression by participating in cell apoptosis, the regulation of cell cycles, DNA repair, cell adhesion, and tissue remodeling [32]. There are three subtypes of CLU. The most widely studied is secretory clu (sCLU), which exists in almost all physiological fluids. Another subtype is nuclear cluster protein (nCLU), which exists in the nucleus. The third subtype is cytoplasmic aggregation protein (cCLU), which mainly exists in the cytoplasm and is still little known [33]. We found that CLU was abundant in serum but not in exosomes, and that CLU expression in lung adenocarcinomas was lower than that in adjacent tissues. According to other scholars’ studies on the expression and potential role of CLU in lung cancer serum and tissue, their results are inconsistent, demonstrating the instability of CLU expression in serum and tissue samples [34]. Even if some studies believe that CLU may be developed into a serological biomarker of lung adenocarcinoma, the serum and tissue samples of CLU are doomed to be difficult to be applied in the clinic. Exosomes are membrane vesicles from endosomes, which can fuse with the plasma membrane and release proteins into the extracellular environment in an insoluble form. Many cancer-related proteins can be secreted from cells to other parts through this non-classical secretory pathway [35]. We detected the expression of CLU in the exosomes of patients with lung adenocarcinoma for the first time. Our excitement was stimulated when we discovered that serum CLU expression in advanced lung adenocarcinoma was significantly higher than that in early lung adenocarcinoma and healthy controls, and the overexpression trend was stable. The idea presents a new clinical application for CLU.

In conclusion, ITGAM and CLU were identified as serum exosomal protein markers of lung adenocarcinoma. This study can serve as a basis for developing treatments for lung adenocarcinomas as a preliminary study.

## Supplementary Information

**Additional file 1: Fig. 1.** The evaluation for the results of protein identification.

**Additional file 2.**

**Additional file 3: Table 1. **The clinical information of individuals providing serum samples.

**Additional file 4: Table 2.** The clinical information of patients providing tissue samples.

**Additional file 5: Table 3. **The raw data from the LC-MS analysis for GSEA.

## Data Availability

The datasets generated and/or analyzed during the current study are publicly available. All data generated or analyzed during this study are included in this published article and its supplementary information files**.**

## Abbreviations

NSCLC: Non-small cell lung cancer
EVs: Extracellular vesicles
ILVs: Intraluminal vesicles
MVBs: Multivesicular endosomes or multivesicular bodies
LC–MS: Liquid chromatography-mass spectrometry
WB: Western blot
IHC: Immunohistochemistry
BCA: Bicinchoninic acid
NTA: Nanoparticle Tracking Analysis
TEM: Transmission Electron Microscopy
SDS-PAGE: Sodium dodecyl sulfate–polyacrylamide gel electrophoresis
HRP: Horseradish peroxidase
ECL: Enhanced chemiluminescence
RPLC: Reversed Phase Liquid Chromatography
HCD: Higher energy collisional dissociation
GSEA: Gene Set Enrichment Analysis
ITGAM: Integrin alpha M chain
CLU: Clusterin
SNP: Single nucleotide polymorphism
NK cells: Natural killer cells
MDSCs: Myeloid-derived suppressor cells
