# Supplementary material for: Serum exosomal proteomics analysis of lung adenocarcinoma to discover new tumor markers
Source: BMC Cancer. 2022 Mar 15;22:279. doi: 10.1186/s12885-022-09366-x (PMC8925168; doi:10.1186/s12885-022-09366-x)
Supplement: Supplementary file 1 — Additional file 1: Fig. 1. The evaluation for the results of protein identification. [file 12885_2022_9366_MOESM1_ESM.docx]

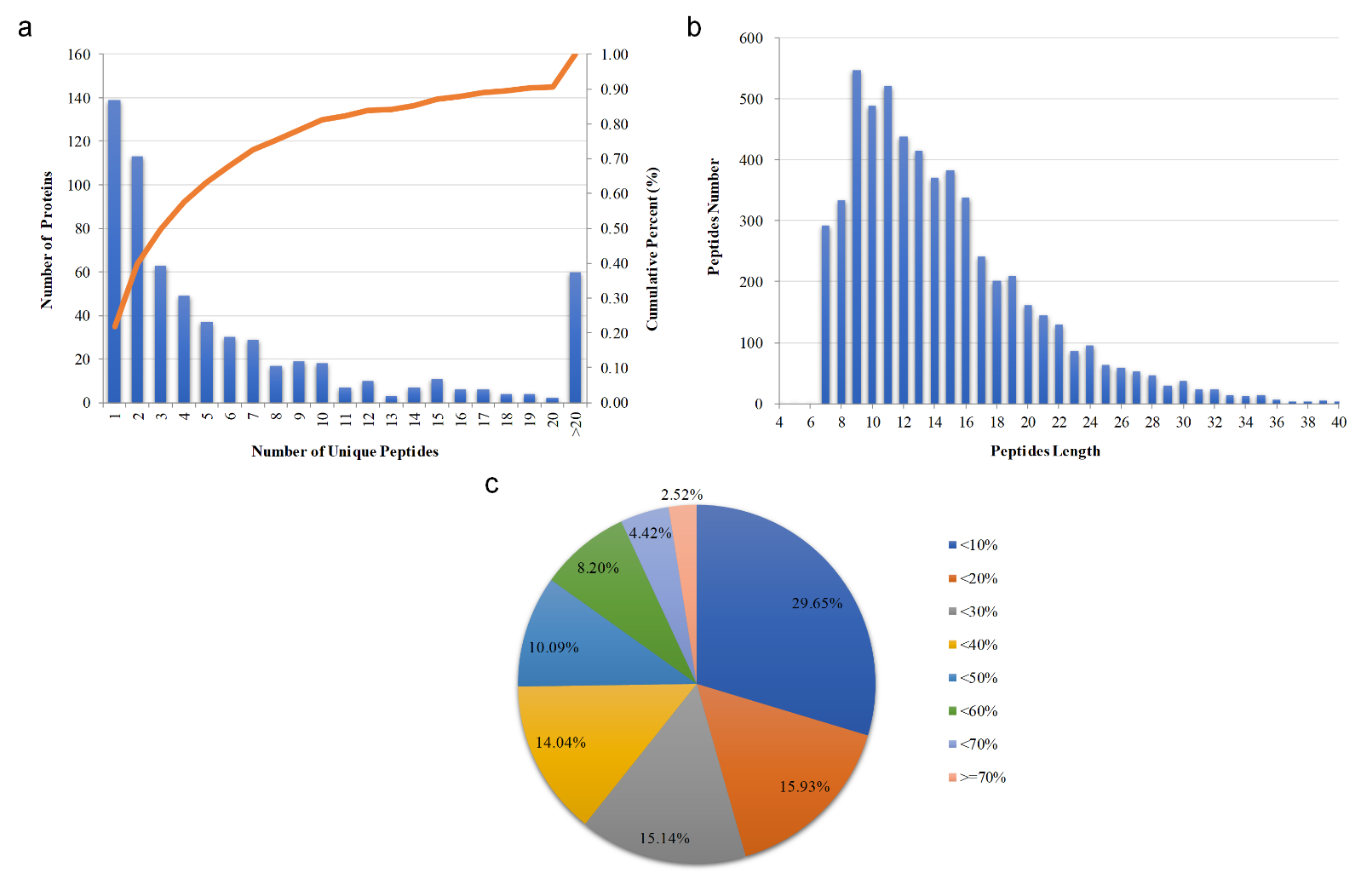


**Supplementary Fig. 1** The evaluation for the results of protein identification. **a** The number distribution of unique peptides. The abscissa represented number of unique peptides contained in proteins. The histogram (x-axis and left y-axis) represented the number of proteins corresponding to different unique peptides. The curve (x-axis and right y-axis) indicated the proportion of the number of proteins corresponding to the cumulative number of unique peptides to the total number of proteins. **b** The distribution of peptides length. **c** The distribution of protein coverage.
