# Supplementary material for: Serum exosomal proteomics analysis of lung adenocarcinoma to discover new tumor markers
Source: BMC Cancer. 2022 Mar 15;22:279. doi: 10.1186/s12885-022-09366-x (PMC8925168; doi:10.1186/s12885-022-09366-x)

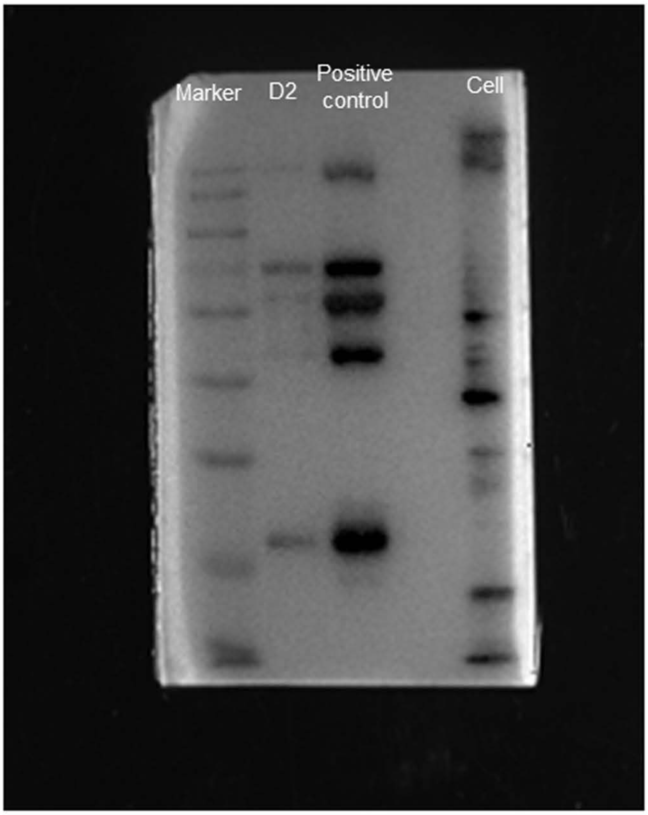

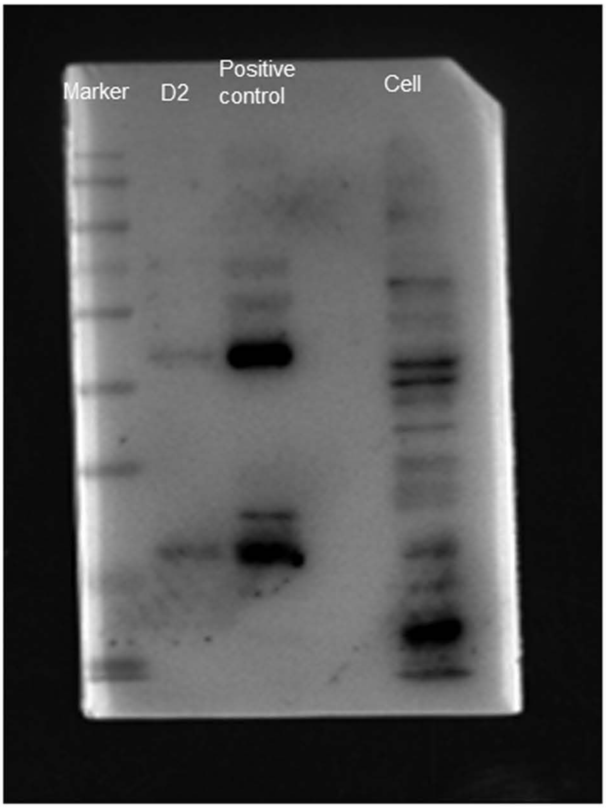

Fig. 1C. Images of the full-length blots. WB results displayed that CD9 and CD81, as exosomal markers, were expressed in isolated exosomes.

27 samples of three groups were used for WB to validate these potentially differential proteins of exosomes (Validation analysis by WB). ①, ②, and ③ referred to the WB grouping. Each group of WB required 3 patients in the advanced lung adenocarcinoma group, early lung adenocarcinoma group, and healthy control group, respectively). Gels/blots of each group cropped from different parts of the same gel. The nonspecific band of ITGAM (130-95 KDa) was overlapped with the band of Alix (96 KDa), therefore, Alix was detected after primary and secondary antibody remover was used to remove the band of ITGAM.

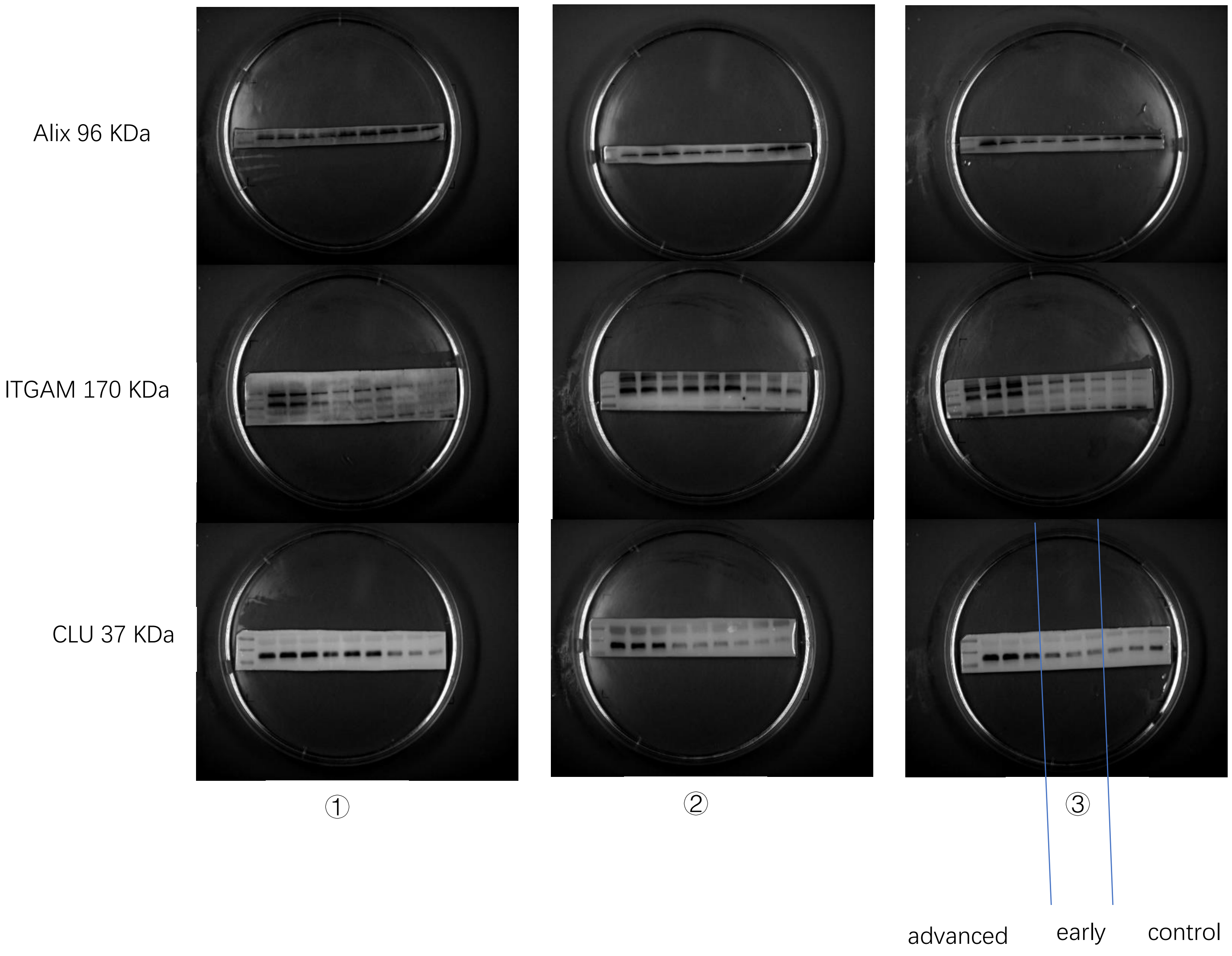

3 samples of the advanced lung adenocarcinoma group were used to make comparisons of different expression models of ITGAM and CLU in exosomes and serum (Comparison analysis by WB). Gels/blots cropped from different parts of the same gel.
Note: initially, we compared two different separation methods (ultracentrifugation and kits). In the paper, we deleted the above content.

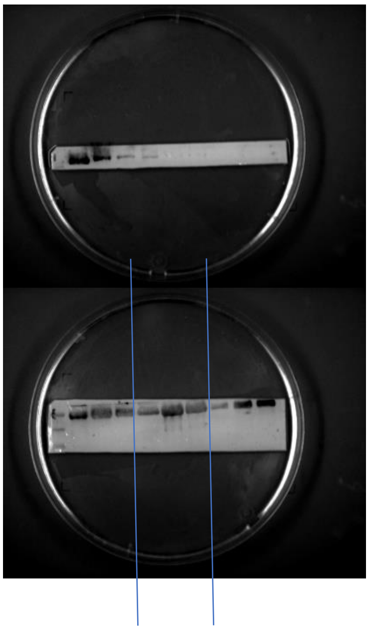

Supplement: Supplementary file 2 — Additional file 2. [file 12885_2022_9366_MOESM2_ESM.pdf]
