# Supplementary material for: Serum exosomal proteomics analysis of lung adenocarcinoma to discover new tumor markers
Source: BMC Cancer. 2022 Mar 15;22:279. doi: 10.1186/s12885-022-09366-x (PMC8925168; doi:10.1186/s12885-022-09366-x)
Supplement: Supplementary file 3 — Additional file 3: Table 1. The clinical information of individuals providing serum samples. [file 12885_2022_9366_MOESM3_ESM.docx]

**Supplementary Table 1** The clinical information of individuals providing serum samples

| Specimen numbering | Age | Gender | Stage | Group | | Experiment |
| --- | --- | --- | --- | --- | --- | --- |
| D1 | 65 | Male | No | Healthy control | | LC-MS |
| D2 | 56 | Male | No |  |  |  |
| D3 | 63 | Female | No |  |  |  |
| 56 | 53 | Male | T2aN1M0，IIA | Early | |  |
| 143 | 67 | Female | T2N0M0，IB |  |  |  |
| 228 | 62 | Male | T1N0M0，ⅠA |  |  |  |
| 37 | 66 | Male | T2N0M1，IV | Advanced | |  |
| 52 | 63 | Male | T2N0M1，IV |  |  |  |
| 65 | 59 | Female | T4N2M1，IV |  |  |  |
| D4 | 63 | Male | No |  | Healthy control | Validation analysis by WB |
| D5 | 52 | Female | No |  |  |  |
| D6 | 49 | Male | No |  |  |  |
| D7 | 70 | Male | No | ② |  |  |
| D8 | 65 | Female | No |  |  |  |
| D9 | 50 | Female | No |  |  |  |
| D10 | 55 | Male | No | ③ |  |  |
| D11 | 68 | Male | No |  |  |  |
| D12 | 62 | Female | No |  |  |  |
| 276 | 54 | Male | T2N0M0，IB |  | Early | Validation analysis by WB |
| 82 | 65 | Male | T2N2M0，IIIA |  |  |  |
| 229 | 48 | Female | T2N0M0，IB |  |  |  |
| 128 | 55 | Male | T2aN1M0，IIA | ② |  |  |
| 300 | 73 | Female | T2aN0M0，IB |  |  |  |
| 356 | 52 | Female | T2N0M0，IB |  |  |  |
| 312 | 53 | Female | T2N2M0，IIIA | ③ |  |  |
| 310 | 67 | Male | T2N1M0，IIB |  |  |  |
| 345 | 64 | Male | T2aN0M0，1B |  |  |  |
| 90 | 68 | Male | T3N0M1b，IV |  | Advanced | Validation analysis by WB |
| 83 | 48 | Female | T2N0M1，IV |  |  |  |
| 102 | 61 | Male | T3N3M1b，IV |  |  |  |
| 36 | 72 | Male | T4N2M0，IIIB | ② |  |  |
| 61 | 63 | Female | T2N0M1，IV |  |  |  |
| 92 | 63 | Female | T4N2M1，IV |  |  |  |
| 127 | 63 | Male | T2aN3M1a，Ⅳ | ③ |  |  |
| 234 | 72 | Female | T1aN1M1b，IV |  |  |  |
| 227 | 77 | Male | T1N0M1，IV |  |  |  |
| 295 | 67 | Male | T4N3M1，IV | Advanced | | Comparison analysis by WB |
| 311 | 61 | Male | T2bN3M0, IIIB |  |  |  |
| 355 | 66 | Male | T3N3M1c，IVB |  |  |  |
