# Supplementary material for: Serum exosomal proteomics analysis of lung adenocarcinoma to discover new tumor markers
Source: BMC Cancer. 2022 Mar 15;22:279. doi: 10.1186/s12885-022-09366-x (PMC8925168; doi:10.1186/s12885-022-09366-x)
Supplement: Supplementary file 4 — Additional file 4: Table 2. The clinical information of patients providing tissue samples. [file 12885_2022_9366_MOESM4_ESM.docx]

**Supplementary Table 2** The clinical information of patients providing tissue samples

| Patient numbering | Sample numbering | Sample type | Age | Gender | Stage | Protein |
| --- | --- | --- | --- | --- | --- | --- |
| 1 | Q-1 | Tumor tissue | 67 | Male | T1bN0M0，IA2 | ITGAM，CLU |
| 2 | Q-2 | Tumor tissue | 64 | Female | T1N1M0，IIB | ITGAM |
| 3 | Q-3 | Tumor tissue | 74 | Female | T1cN0M0，IA3 | ITGAM，CLU |
| 4 | Q-4 | Tumor tissue | 60 | Female | T1cN0M0，IA3 | ITGAM，CLU |
| 5 | Q-5 | Tumor tissue | 55 | Female | T1cN2M0，IIIA | ITGAM，CLU |
| 6 | Q-6 | Tumor tissue | 68 | Male | T1N2Mx，IIIA | ITGAM，CLU |
| 7 | Q-7 | Tumor tissue | 67 | Male | T3N0M0，IIB | ITGAM，CLU |
| 8 | Q-8 | Tumor tissue | 52 | Female | T1cN0M0，IA3 | ITGAM，CLU |
| 9 | Q-9 | Tumor tissue | 37 | Male | T1cN0M0，IA3 | ITGAM，CLU |
| 10 | Q-10 | Tumor tissue | 62 | Male | T2N0M0，II | ITGAM，CLU |
| 11 | Q-11 | Tumor tissue | 65 | Male | T1cN2M0，IIIA | ITGAM，CLU |
| 12 | Q-12 | Tumor tissue | 48 | Male | T4N2M0，IIIB | ITGAM，CLU |
| 13 | Q-13 | Tumor tissue | 61 | Male | T1N0M0，IA | ITGAM |
| 14 | Q-14 | Tumor tissue | 56 | Male | T1aN0M0，IA | ITGAM，CLU |
| 15 | Q-15 | Tumor tissue | 68 | Male | T1N2Mx，IIIA | ITGAM，CLU |
| 1 | Q-16 | Adjacent tissue | 67 | Male | T1bN0M0，IA2 | CLU |
| 4 | Q-17 | Adjacent tissue | 60 | Female | T1cN0M0，IA3 | ITGAM，CLU |
| 8 | Q-18 | Adjacent tissue | 52 | Female | T1cN0M0，IA3 | ITGAM，CLU |
| 9 | Q-19 | Adjacent tissue | 37 | Male | T1cN0M0，IA3 | ITGAM，CLU |
| 10 | Q-20 | Adjacent tissue | 62 | Male | T2N0M0，II | ITGAM，CLU |
