# Supplementary material for: Serum exosomal proteomics analysis of lung adenocarcinoma to discover new tumor markers
Source: BMC Cancer. 2022 Mar 15;22:279. doi: 10.1186/s12885-022-09366-x (PMC8925168; doi:10.1186/s12885-022-09366-x)
Supplement: Supplementary file 5 — Additional file 5: Table 3. The raw data from the LC-MS analysis for GSEA. [file 12885_2022_9366_MOESM5_ESM.docx]

**Supplementary Table 3** The raw data from the LC-MS analysis for GSEA

| Gene names | description | Healthy control group | | | Early lung adenocarcinoma group | | | Advanced lung adenocarcinoma group | | |
| --- | --- | --- | --- | --- | --- | --- | --- | --- | --- | --- |
|  |  | D1 | D2 | D3 | 56 | 143 | 228 | 37 | 52 | 65 |
| ZNF74 | na | 1.06E+08 | 0 | 0 | 0 | 0 | 0 | 0 | 0 | 0 |
| YWHAZ | na | 3.31E+09 | 37142000 | 8.95E+08 | 1.14E+09 | 1.11E+09 | 1.08E+09 | 4.9E+08 | 4.97E+08 | 2.54E+08 |
| YWHAH | na | 36656000 | 0 | 19891000 | 41187000 | 56855000 | 89480000 | 0 | 0 | 0 |
| YWHAG | na | 0 | 0 | 0 | 0 | 0 | 0 | 0 | 0 | 0 |
| YWHAE | na | 1.23E+08 | 0 | 30880000 | 44645000 | 36655000 | 27244000 | 17788000 | 28353000 | 13548000 |
| YWHAB | na | 61437000 | 0 | 0 | 0 | 18261000 | 0 | 0 | 0 | 0 |
| XPNPEP2 | na | 0 | 0 | 1.48E+08 | 0 | 0 | 1869300 | 0 | 0 | 0 |
| XP32 | na | 0 | 0 | 0 | 0 | 0 | 0 | 0 | 9469200 | 0 |
| WHAMM | na | 3.5E+08 | 7.03E+08 | 8.02E+08 | 5.6E+08 | 2.28E+08 | 0 | 6.1E+08 | 56991000 | 0 |
| WDR1 | na | 5.31E+08 | 0 | 1.55E+08 | 1.45E+08 | 28872000 | 1.17E+08 | 78195000 | 18520000 | 0 |
| WARS | na | 0 | 0 | 0 | 0 | 0 | 0 | 0 | 0 | 0 |
| VWF | na | 3.75E+09 | 1.6E+09 | 1.2E+10 | 5.49E+09 | 4.49E+09 | 5.27E+09 | 5.01E+09 | 2.81E+10 | 1.36E+10 |
| VTN | na | 2.65E+09 | 1.17E+10 | 4.19E+09 | 1.2E+10 | 7.98E+09 | 9.23E+09 | 8.71E+09 | 9.46E+09 | 1.01E+10 |
| VTI1B | na | 0 | 0 | 0 | 45505000 | 0 | 0 | 0 | 0 | 0 |
| VPS13A | na | 4.72E+08 | 0 | 4.8E+08 | 1.22E+08 | 2.6E+08 | 0 | 0 | 0 | 3.07E+08 |
| VCP | na | 16245000 | 0 | 64012000 | 37765000 | 98661000 | 1.56E+08 | 18994000 | 69817000 | 92025000 |
| VCL | na | 1.12E+09 | 0 | 2.02E+08 | 1.7E+08 | 1.81E+08 | 1.51E+08 | 36225000 | 55423000 | 48862000 |
| VCAN | na | 41559000 | 0 | 0 | 0 | 0 | 0 | 0 | 28453000 | 0 |
| VASP | na | 85438000 | 0 | 23363000 | 81647000 | 34672000 | 2.51E+08 | 14019000 | 64644000 | 62611000 |
| UMOD | na | 0 | 29686000 | 1.32E+08 | 21279000 | 6.38E+08 | 1.01E+08 | 0 | 0 | 19038000 |
| UGP2 | na | 0 | 0 | 10184000 | 0 | 0 | 44232000 | 0 | 0 | 0 |
| UGGT1 | na | 0 | 0 | 11849000 | 6491200 | 59400000 | 0 | 19280000 | 0 | 63584000 |
| UBAC2 | na | 7.52E+08 | 42659000 | 3.12E+08 | 0 | 0 | 0 | 1.3E+08 | 1.33E+08 | 2.43E+08 |
| UBA52 | na | 4.3E+08 | 93453000 | 4.38E+08 | 1.29E+08 | 3.07E+08 | 2.33E+08 | 2.15E+08 | 2.27E+08 | 4.58E+08 |
| TUBB | na | 2.11E+08 | 5088200 | 1.02E+08 | 83321000 | 49185000 | 54670000 | 61983000 | 37355000 | 13415000 |
| TUBA4A | na | 8.92E+08 | 0 | 2.48E+08 | 1.33E+08 | 92438000 | 1.11E+08 | 30539000 | 32859000 | 10986000 |
| TUBA1C | na | 46956000 | 0 | 14675000 | 0 | 7026000 | 0 | 0 | 0 | 0 |
| TUBA1B | na | 1.97E+08 | 0 | 42232000 | 36760000 | 35254000 | 30774000 | 25061000 | 24644000 | 23979000 |
| TTYH3 | na | 1.6E+08 | 27035000 | 1.08E+08 | 94210000 | 1.41E+08 | 1.11E+08 | 69325000 | 1.05E+08 | 88194000 |
| TTR | na | 2.77E+08 | 4.82E+09 | 5.02E+08 | 1.43E+09 | 9.88E+08 | 1.26E+09 | 1.7E+09 | 1.48E+09 | 3.48E+08 |
| TSPAN9 | na | 3.66E+08 | 27053000 | 1.2E+08 | 89832000 | 99254000 | 87959000 | 1.04E+08 | 82356000 | 77230000 |
| TSPAN4 | na | 0 | 0 | 4730000 | 0 | 6153000 | 13005000 | 8361500 | 0 | 8653000 |
| TSPAN33 | na | 23196000 | 9831800 | 66161000 | 29390000 | 1.07E+08 | 67427000 | 18435000 | 0 | 0 |
| TSPAN14 | na | 1.65E+08 | 40716000 | 1.47E+08 | 1.16E+08 | 2.23E+08 | 1.97E+08 | 1.13E+08 | 89300000 | 77378000 |
| TRRAP | na | 0 | 0 | 0 | 0 | 0 | 0 | 0 | 0 | 0 |
| TRIM38 | na | 49454000 | 0 | 70024000 | 0 | 42170000 | 24139000 | 0 | 0 | 0 |
| TNXB | na | 0 | 35177000 | 0 | 0 | 0 | 0 | 0 | 0 | 0 |
| TNC | na | 34193000 | 0 | 13390000 | 11900000 | 16856000 | 0 | 0 | 85209000 | 0 |
| TMSB4X | na | 1.32E+08 | 15136000 | 53072000 | 41194000 | 41358000 | 43930000 | 0 | 58463000 | 0 |
| TMEM198 | na | 55426000 | 1.65E+09 | 1.26E+08 | 3.52E+08 | 3.7E+08 | 2E+08 | 3.1E+08 | 1.61E+08 | 0 |
| TMBIM1 | na | 67547000 | 0 | 26217000 | 26627000 | 68333000 | 30667000 | 30075000 | 33625000 | 40444000 |
| TLN1 | na | 7.08E+09 | 3342700 | 2.18E+09 | 2.02E+09 | 2.17E+09 | 1.93E+09 | 1.04E+09 | 8.79E+08 | 1.59E+09 |
| THBS4 | na | 0 | 0 | 0 | 0 | 0 | 17308000 | 0 | 13043000 | 49486000 |
| TGFBI | na | 0 | 15303000 | 11873000 | 10190000 | 7014000 | 12221000 | 8595200 | 10531000 | 7280200 |
| TGFB1I1 | na | 3390400 | 0 | 0 | 0 | 3713800 | 2501300 | 0 | 0 | 0 |
| TGFB1 | na | 10198000 | 0 | 0 | 0 | 0 | 0 | 0 | 0 | 0 |
| TFRC | na | 1.83E+09 | 2.97E+08 | 5.05E+08 | 7.08E+08 | 3.64E+08 | 1.04E+09 | 2.91E+08 | 1.28E+09 | 8.98E+08 |
| TFG | na | 60203000 | 0 | 31686000 | 0 | 0 | 0 | 0 | 0 | 0 |
| TF | na | 1.76E+10 | 2.89E+11 | 4.42E+10 | 5.74E+10 | 4.87E+10 | 6.75E+10 | 6.44E+10 | 6.15E+10 | 1.15E+10 |
| TAGLN2 | na | 2.75E+08 | 0 | 93079000 | 78871000 | 1.07E+08 | 82187000 | 45146000 | 55463000 | 20784000 |
| SVOP | na | 3.87E+08 | 0 | 0 | 23408000 | 9596800 | 0 | 95499000 | 0 | 0 |
| SVEP1 | na | 84387000 | 44449000 | 1.16E+08 | 38688000 | 28235000 | 14166000 | 12548000 | 48027000 | 70428000 |
| SUSD1 | na | 4326300 | 0 | 0 | 0 | 2913100 | 4657500 | 0 | 0 | 0 |
| STXBP5 | na | 0 | 1.07E+09 | 0 | 3.06E+09 | 0 | 1.08E+09 | 0 | 1.57E+09 | 0 |
| STXBP2 | na | 16149000 | 0 | 20209000 | 6050000 | 4741700 | 18308000 | 6172100 | 12521000 | 4417600 |
| STX7 | na | 10566000 | 0 | 15459000 | 22544000 | 21197000 | 16241000 | 0 | 9289300 | 0 |
| STOM | na | 4.45E+09 | 3.42E+08 | 1.77E+09 | 2.62E+09 | 4.28E+09 | 3.05E+09 | 1.09E+09 | 2.03E+09 | 1.55E+09 |
| STEAP3 | na | 27626000 | 0 | 7120300 | 12023000 | 11662000 | 23824000 | 7708000 | 22721000 | 32026000 |
| STAM | na | 4.08E+08 | 48108000 | 3.64E+08 | 2E+08 | 87453000 | 1.22E+08 | 2.16E+08 | 1.34E+08 | 3.92E+08 |
| ST8SIA4 | na | 0 | 0 | 17077000 | 0 | 16908000 | 0 | 17990000 | 0 | 0 |
| SSC5D | na | 19858000 | 0 | 0 | 9484900 | 0 | 10527000 | 41074000 | 11286000 | 47682000 |
| SRI | na | 5.21E+08 | 1.6E+08 | 5.1E+08 | 8.17E+08 | 1.34E+09 | 1.27E+09 | 4.69E+08 | 6.1E+08 | 4.43E+08 |
| SRC | na | 59583000 | 0 | 15127000 | 0 | 33313000 | 13703000 | 0 | 0 | 0 |
| SPTBN1 | na | 6.09E+08 | 0 | 3.75E+08 | 2.46E+08 | 2.18E+08 | 2.67E+08 | 1.88E+08 | 1.35E+08 | 4.96E+08 |
| SPP2 | na | 0 | 30587000 | 0 | 26578000 | 87018000 | 57491000 | 36134000 | 45973000 | 55425000 |
| SORD | na | 0 | 0 | 0 | 0 | 0 | 0 | 0 | 13569000 | 55743000 |
| SNAP23 | na | 38869000 | 0 | 0 | 0 | 19862000 | 17282000 | 0 | 0 | 0 |
| SMIM5 | na | 25536000 | 5139500 | 16799000 | 12598000 | 27546000 | 17484000 | 9795900 | 7616300 | 13209000 |
| SLC4A1 | na | 5.05E+08 | 1.17E+08 | 6.73E+08 | 3.81E+09 | 2.59E+09 | 2.44E+09 | 3.24E+08 | 3.08E+09 | 7.27E+08 |
| SLC44A2 | na | 1.89E+08 | 0 | 1.65E+08 | 1.83E+08 | 2.72E+08 | 1.42E+08 | 1.37E+08 | 89139000 | 1.76E+08 |
| SLC44A1 | na | 2.46E+08 | 39959000 | 1.8E+08 | 2.06E+08 | 3.13E+08 | 1.92E+08 | 1.52E+08 | 97062000 | 1.13E+08 |
| SLC2A3 | na | 2.75E+08 | 23429000 | 60730000 | 73901000 | 89455000 | 52192000 | 42919000 | 53044000 | 46460000 |
| SLC2A1 | na | 1.2E+08 | 0 | 1.3E+08 | 3.61E+08 | 2.54E+08 | 2.35E+08 | 55251000 | 3.38E+08 | 1.77E+08 |
| SLC29A1 | na | 28962000 | 0 | 19656000 | 44668000 | 30647000 | 30380000 | 0 | 43207000 | 26610000 |
| SLC25A19 | na | 0 | 0 | 1.73E+08 | 0 | 0 | 0 | 0 | 23587000 | 40696000 |
| SLC1A5 | na | 9987100 | 0 | 3372000 | 1154800 | 4676200 | 3767300 | 0 | 6862000 | 0 |
| SKAP2 | na | 39116000 | 0 | 7398700 | 0 | 0 | 0 | 0 | 0 | 0 |
| SIRPA | na | 9.57E+08 | 1.33E+08 | 1.15E+08 | 1.91E+08 | 2.08E+08 | 1.29E+08 | 1.04E+09 | 1.53E+08 | 3.4E+08 |
| SIGLEC16 | na | 80017000 | 1.23E+08 | 0 | 2.1E+08 | 4.82E+08 | 0 | 77828000 | 3.1E+08 | 0 |
| SHBG | na | 4073400 | 23958000 | 0 | 3175900 | 5178600 | 3114400 | 3550400 | 5322800 | 0 |
| SH3BGRL3 | na | 78803000 | 0 | 12236000 | 0 | 0 | 0 | 0 | 0 | 0 |
| SFTPA1 | na | 0 | 0 | 0 | 0 | 11028000 | 0 | 0 | 0 | 0 |
| SERPING1 | na | 1.4E+09 | 6.5E+09 | 2.09E+09 | 2.6E+09 | 2.51E+09 | 1.98E+09 | 2.62E+09 | 2.81E+09 | 2E+09 |
| SERPINF2 | na | 4.62E+08 | 2.16E+09 | 5.06E+08 | 6.27E+08 | 5.43E+08 | 7.5E+08 | 8.12E+08 | 8.61E+08 | 5.38E+08 |
| SERPINF1 | na | 0 | 5.23E+08 | 45012000 | 84674000 | 1.19E+08 | 1.73E+08 | 96644000 | 1.9E+08 | 0 |
| SERPIND1 | na | 4.61E+08 | 2.43E+09 | 6.9E+08 | 8.05E+08 | 5.76E+08 | 5.62E+08 | 5.51E+08 | 1.15E+09 | 3.73E+08 |
| SERPINC1 | na | 1.93E+09 | 7.71E+09 | 3.07E+09 | 5.37E+09 | 3.73E+09 | 4.97E+09 | 5.72E+09 | 5.95E+09 | 4.73E+09 |
| SERPINB1 | na | 13700000 | 0 | 0 | 0 | 5260800 | 0 | 0 | 0 | 0 |
| SERPINA7 | na | 0 | 4.56E+08 | 0 | 14414000 | 20579000 | 39919000 | 10998000 | 35431000 | 3854800 |
| SERPINA6 | na | 0 | 8.8E+08 | 20095000 | 73055000 | 60617000 | 60665000 | 61305000 | 71267000 | 0 |
| SERPINA5 | na | 1.98E+08 | 2.3E+08 | 2.73E+08 | 3.07E+08 | 2.26E+08 | 2.71E+08 | 2.46E+08 | 2.67E+08 | 2.83E+08 |
| SERPINA4 | na | 1.44E+08 | 7.92E+08 | 1.04E+08 | 3.15E+08 | 2.32E+08 | 3.73E+08 | 3.39E+08 | 1.97E+08 | 1.06E+08 |
| SERPINA3 | na | 5.92E+08 | 8.23E+09 | 7.24E+08 | 2.2E+09 | 1.83E+09 | 2.09E+09 | 1.44E+09 | 3.83E+09 | 8.39E+08 |
| SERPINA10 | na | 14908000 | 41624000 | 21120000 | 16350000 | 16110000 | 0 | 25453000 | 20932000 | 0 |
| SERPINA1 | na | 3.59E+09 | 8.82E+10 | 9.05E+09 | 1.59E+10 | 1.94E+10 | 2.06E+10 | 1.68E+10 | 2.28E+10 | 5.39E+09 |
| SELL | na | 0 | 30274000 | 0 | 0 | 18338000 | 23012000 | 0 | 0 | 0 |
| SELENOP | na | 1.14E+08 | 82251000 | 54494000 | 83640000 | 73176000 | 65156000 | 0 | 36238000 | 55726000 |
| SDCBP | na | 1.68E+08 | 14460000 | 1.07E+08 | 1.15E+08 | 2.12E+08 | 1.89E+08 | 90895000 | 1.06E+08 | 2.79E+08 |
| SAA4 | na | 22263000 | 1.54E+08 | 63518000 | 78446000 | 67543000 | 54154000 | 41170000 | 1.17E+08 | 4457300 |
| SAA1 | na | 0 | 45121000 | 48118000 | 8525200 | 6745600 | 9381100 | 6306400 | 2.58E+08 | 0 |
| S100A9 | na | 23099000 | 54784000 | 2.53E+08 | 96472000 | 1.73E+08 | 2.93E+08 | 2E+08 | 4.84E+08 | 6.07E+08 |
| S100A8 | na | 0 | 34843000 | 95107000 | 0 | 1.24E+08 | 1.57E+08 | 0 | 1.4E+08 | 1.57E+08 |
| S100A7A | na | 0 | 0 | 30824000 | 7372200 | 0 | 0 | 0 | 6010800 | 0 |
| S100A12 | na | 0 | 0 | 0 | 2463100 | 0 | 0 | 0 | 4659000 | 3971600 |
| S100A11 | na | 0 | 0 | 0 | 0 | 7875600 | 0 | 0 | 6758000 | 0 |
| RSU1 | na | 3.6E+08 | 0 | 1.21E+08 | 1.14E+08 | 1.3E+08 | 1.06E+08 | 66712000 | 47442000 | 0 |
| RHD | na | 4169200 | 0 | 7671200 | 25815000 | 28735000 | 19164000 | 6056600 | 20560000 | 12763000 |
| RHAG | na | 0 | 0 | 0 | 5827800 | 10159000 | 0 | 0 | 7145900 | 0 |
| RETN | na | 1.29E+08 | 0 | 0 | 0 | 44520000 | 27548000 | 0 | 14450000 | 68469000 |
| RELN | na | 1.38E+08 | 83832000 | 1.31E+08 | 1.59E+08 | 74016000 | 39125000 | 0 | 67053000 | 72489000 |
| RBP4 | na | 0 | 2.19E+09 | 88792000 | 1.15E+09 | 9.69E+08 | 8.67E+08 | 9.25E+08 | 8.68E+08 | 0 |
| RASA3 | na | 11446000 | 0 | 0 | 0 | 3326400 | 3269200 | 0 | 0 | 0 |
| RAP2B | na | 50446000 | 0 | 0 | 20301000 | 28097000 | 0 | 0 | 16044000 | 19657000 |
| RAP1B | na | 7.36E+08 | 1.28E+08 | 1.46E+08 | 6.62E+08 | 6.35E+08 | 7.25E+08 | 4.26E+08 | 5.68E+08 | 2.73E+08 |
| RANBP3L | na | 3.57E+08 | 0 | 92961000 | 1.41E+08 | 3.21E+08 | 0 | 1.19E+08 | 0 | 2.24E+08 |
| RAC2 | na | 37411000 | 0 | 0 | 26561000 | 54592000 | 41124000 | 0 | 28116000 | 20465000 |
| RAB7A | na | 80308000 | 0 | 0 | 29105000 | 41572000 | 36764000 | 23640000 | 37456000 | 35229000 |
| RAB6B | na | 90291000 | 0 | 38146000 | 21867000 | 69002000 | 35849000 | 18159000 | 40099000 | 17806000 |
| RAB27B | na | 68572000 | 40601000 | 37556000 | 57870000 | 59899000 | 38198000 | 42553000 | 24175000 | 36617000 |
| RAB1B | na | 0 | 0 | 0 | 0 | 15289000 | 0 | 0 | 0 | 0 |
| RAB14 | na | 0 | 0 | 0 | 10209000 | 16230000 | 11061000 | 0 | 0 | 14851000 |
| RAB11A | na | 23285000 | 0 | 9693600 | 0 | 14111000 | 0 | 0 | 10292000 | 0 |
| RAB10 | na | 2.28E+08 | 0 | 49114000 | 48084000 | 76272000 | 64845000 | 35339000 | 47067000 | 64770000 |
| QSOX1 | na | 0 | 9565500 | 0 | 0 | 0 | 0 | 0 | 0 | 0 |
| PZP | na | 72695000 | 2.19E+08 | 83494000 | 0 | 1.27E+08 | 88195000 | 3069700 | 1.52E+09 | 4.63E+08 |
| PXDN | na | 1.46E+08 | 0 | 3846400 | 0 | 1015400 | 882200 | 0 | 0 | 0 |
| PTX3 | na | 0 | 0 | 0 | 14553000 | 2.41E+08 | 71010000 | 7511800 | 1.17E+08 | 1.25E+08 |
| PTTG1IP | na | 24907000 | 0 | 18674000 | 16670000 | 19476000 | 13351000 | 11492000 | 16126000 | 11046000 |
| PTPRJ | na | 1.22E+08 | 0 | 46432000 | 23304000 | 20123000 | 26247000 | 18543000 | 16693000 | 15087000 |
| PTPRC | na | 80905000 | 0 | 45167000 | 26889000 | 26793000 | 15955000 | 9264500 | 7488000 | 73521000 |
| PTGDS | na | 0 | 0 | 0 | 0 | 13544000 | 0 | 0 | 0 | 0 |
| PSMB6 | na | 0 | 0 | 54222000 | 0 | 0 | 0 | 0 | 27614000 | 0 |
| PSMB5 | na | 0 | 0 | 61503000 | 0 | 0 | 0 | 0 | 34185000 | 0 |
| PSMB4 | na | 0 | 0 | 83851000 | 31935000 | 0 | 0 | 16621000 | 47191000 | 0 |
| PSMB3 | na | 0 | 0 | 16874000 | 0 | 0 | 0 | 0 | 10142000 | 0 |
| PSMB2 | na | 0 | 0 | 0 | 0 | 0 | 0 | 0 | 0 | 0 |
| PSMB1 | na | 23508000 | 0 | 79140000 | 14459000 | 0 | 22066000 | 17036000 | 43845000 | 34349000 |
| PSMA7 | na | 0 | 0 | 71759000 | 4747300 | 7802800 | 0 | 0 | 32751000 | 6468600 |
| PSMA6 | na | 32878000 | 0 | 1.31E+08 | 29584000 | 31763000 | 23695000 | 33074000 | 1.01E+08 | 49277000 |
| PSMA5 | na | 0 | 0 | 95209000 | 0 | 0 | 0 | 33398000 | 60842000 | 30718000 |
| PSMA4 | na | 59918000 | 0 | 1.06E+08 | 41331000 | 39806000 | 16054000 | 34305000 | 56792000 | 29088000 |
| PSMA2 | na | 7964600 | 0 | 1.15E+08 | 16038000 | 8163300 | 6516800 | 9303600 | 35478000 | 25663000 |
| PSMA1 | na | 0 | 0 | 1.14E+08 | 31383000 | 10768000 | 0 | 0 | 43647000 | 16825000 |
| PRTN3 | na | 0 | 0 | 0 | 0 | 0 | 0 | 7675000 | 8914100 | 8453800 |
| PROZ | na | 0 | 21138000 | 8827600 | 18183000 | 11688000 | 0 | 0 | 0 | 0 |
| PROS1 | na | 9.58E+09 | 3.12E+09 | 1.32E+10 | 6.7E+09 | 4.84E+09 | 5.02E+09 | 9.75E+09 | 8.41E+09 | 1.26E+10 |
| PROC | na | 0 | 41337000 | 0 | 0 | 0 | 0 | 0 | 2856000 | 0 |
| PRG4 | na | 0 | 26244000 | 0 | 8405900 | 9502700 | 0 | 0 | 12363000 | 0 |
| PRDX6 | na | 96545000 | 0 | 22295000 | 16535000 | 35396000 | 18706000 | 0 | 29551000 | 11419000 |
| PRDX2 | na | 1.17E+09 | 0 | 62269000 | 2.35E+08 | 1.98E+08 | 1.02E+08 | 3.29E+08 | 1.4E+08 | 1.21E+08 |
| PPIB | na | 4814800 | 0 | 0 | 4324800 | 10767000 | 0 | 0 | 8720700 | 0 |
| PPIA | na | 6.97E+08 | 0 | 2.76E+08 | 2.34E+08 | 3.17E+08 | 2.42E+08 | 40491000 | 1.44E+08 | 65636000 |
| PPBP | na | 4.63E+08 | 1.2E+09 | 2.23E+08 | 3.22E+08 | 2.85E+08 | 1.46E+08 | 5.02E+08 | 3.46E+08 | 56661000 |
| PON3 | na | 0 | 9718900 | 0 | 0 | 0 | 0 | 0 | 0 | 0 |
| PON1 | na | 2.92E+08 | 2.32E+09 | 2.86E+08 | 7.05E+08 | 4.15E+08 | 6.11E+08 | 6.44E+08 | 4.54E+08 | 2.33E+08 |
| PM20D1 | na | 0 | 4303700 | 9850300 | 0 | 0 | 0 | 0 | 0 | 0 |
| PLTP | na | 30227000 | 42142000 | 35570000 | 13934000 | 40466000 | 33776000 | 0 | 10092000 | 21759000 |
| PLG | na | 1.73E+09 | 1.78E+10 | 2.38E+09 | 8.1E+09 | 5.21E+09 | 6.4E+09 | 7.64E+09 | 6.87E+09 | 2.07E+09 |
| PLEK | na | 5.54E+08 | 72724000 | 2.43E+08 | 1.93E+08 | 2.22E+08 | 2.65E+08 | 1.86E+08 | 1.66E+08 | 53989000 |
| PLCG2 | na | 99294000 | 50346000 | 0 | 93288000 | 0 | 1.44E+08 | 1.08E+08 | 68807000 | 60803000 |
| PLAC8 | na | 0 | 0 | 11084000 | 0 | 0 | 11029000 | 0 | 7321700 | 8284500 |
| PKM | na | 77450000 | 0 | 46432000 | 34904000 | 28479000 | 0 | 0 | 24459000 | 0 |
| PIGR | na | 4.07E+09 | 3.67E+08 | 2.48E+09 | 2.03E+09 | 5.69E+09 | 1.28E+09 | 2.69E+09 | 7.64E+08 | 1.07E+10 |
| PGLYRP2 | na | 1.24E+08 | 6.39E+08 | 78410000 | 1.96E+08 | 1.76E+08 | 1.93E+08 | 1.82E+08 | 2.16E+08 | 84511000 |
| PGK1 | na | 52993000 | 0 | 19979000 | 0 | 0 | 0 | 0 | 18277000 | 17669000 |
| PFN1 | na | 6.7E+08 | 28381000 | 1.88E+08 | 1.77E+08 | 2.45E+08 | 1.79E+08 | 81744000 | 95726000 | 82117000 |
| PF4V1 | na | 3.98E+08 | 20830000 | 1.26E+08 | 42969000 | 44961000 | 1.27E+08 | 14554000 | 37780000 | 0 |
| PF4 | na | 5.25E+10 | 2.28E+09 | 2.71E+10 | 9.34E+09 | 1.23E+10 | 1.78E+10 | 5.57E+09 | 1.17E+10 | 3.21E+09 |
| PEF1 | na | 13081000 | 0 | 3575200 | 22784000 | 47260000 | 37783000 | 0 | 30208000 | 13083000 |
| PECAM1 | na | 4.97E+08 | 0 | 58926000 | 52251000 | 1.24E+08 | 50819000 | 37851000 | 29171000 | 0 |
| PDIA3 | na | 0 | 0 | 6075500 | 5894000 | 9460700 | 5989300 | 3857600 | 6049600 | 4883700 |
| PDCD6IP | na | 26044000 | 0 | 23965000 | 17774000 | 27111000 | 17160000 | 0 | 21383000 | 28611000 |
| PDCD6 | na | 26718000 | 9141400 | 10011000 | 39930000 | 94292000 | 74459000 | 29720000 | 1.59E+08 | 32037000 |
| PCYOX1 | na | 0 | 0 | 0 | 0 | 0 | 0 | 0 | 0 | 0 |
| PCDHB12 | na | 0 | 0 | 0 | 0 | 0 | 0 | 1.05E+09 | 0 | 0 |
| PCCB | na | 0 | 0 | 1.81E+08 | 0 | 0 | 0 | 0 | 0 | 0 |
| PCCA | na | 0 | 0 | 3.7E+08 | 0 | 13516000 | 0 | 0 | 0 | 0 |
| PARVB | na | 5.41E+08 | 0 | 2.17E+08 | 2.16E+08 | 1.66E+08 | 1.96E+08 | 75703000 | 46830000 | 26446000 |
| P4HB | na | 19785000 | 0 | 16712000 | 15971000 | 30000000 | 15691000 | 8888700 | 18240000 | 27742000 |
| ORM2 | na | 1.35E+09 | 7.81E+09 | 1.21E+09 | 2.05E+09 | 1.11E+09 | 1.58E+09 | 2.62E+09 | 2.08E+09 | 1.91E+09 |
| ORM1 | na | 5.45E+08 | 2.12E+10 | 1.28E+09 | 2.79E+09 | 2.52E+09 | 2.39E+09 | 1.78E+09 | 4.9E+09 | 8.06E+08 |
| OLFM4 | na | 10867000 | 14991000 | 4.59E+08 | 1.47E+08 | 4.05E+08 | 1.42E+09 | 0 | 6.69E+08 | 2.26E+09 |
| OIT3 | na | 38684000 | 0 | 24320000 | 0 | 15057000 | 12472000 | 20101000 | 26863000 | 0 |
| NT5E | na | 0 | 30084000 | 1.31E+08 | 11223000 | 0 | 0 | 0 | 0 | 0 |
| NIF3L1 | na | 2.23E+09 | 0 | 0 | 0 | 0 | 594750 | 3.28E+08 | 4.67E+08 | 1881100 |
| NFE2L1 | na | 1.74E+08 | 50264000 | 1.24E+08 | 28038000 | 47836000 | 52942000 | 1.13E+08 | 0 | 54939000 |
| NAPA | na | 0 | 0 | 13134000 | 15928000 | 30429000 | 28251000 | 19512000 | 28639000 | 0 |
| MYL6 | na | 1.68E+08 | 0 | 88079000 | 1.12E+08 | 3.57E+08 | 2.07E+08 | 19685000 | 1.11E+08 | 4.05E+08 |
| MYL12A | na | 1.19E+08 | 0 | 44975000 | 28908000 | 2.8E+08 | 55017000 | 4483500 | 69340000 | 3.04E+08 |
| MYH9 | na | 7.1E+08 | 0 | 1.5E+08 | 2.91E+08 | 1.28E+09 | 4.13E+08 | 31875000 | 6.61E+08 | 1.5E+09 |
| MYH13 | na | 0 | 9.33E+08 | 39494000 | 70344000 | 53283000 | 1.84E+08 | 81376000 | 76230000 | 0 |
| MYCT1 | na | 1.12E+08 | 0 | 34788000 | 88709000 | 51843000 | 19509000 | 1.18E+08 | 28710000 | 0 |
| MYADM | na | 50990000 | 14864000 | 48516000 | 48965000 | 87390000 | 67944000 | 31586000 | 31663000 | 35799000 |
| MVP | na | 0 | 0 | 0 | 3997600 | 6302900 | 4038000 | 0 | 2997700 | 5171900 |
| MTPN | na | 18405000 | 0 | 2620600 | 5571700 | 3471000 | 2698400 | 0 | 3364800 | 0 |
| MTCH1 | na | 42116000 | 0 | 0 | 0 | 14659000 | 0 | 38315000 | 4542800 | 55127000 |
| MST1 | na | 59258000 | 1.37E+08 | 0 | 49453000 | 35810000 | 48786000 | 37179000 | 51828000 | 34005000 |
| MSN | na | 2.02E+08 | 14559000 | 85246000 | 72771000 | 73377000 | 56364000 | 31307000 | 39604000 | 60794000 |
| MRC1 | na | 0 | 2.42E+08 | 0 | 0 | 61611000 | 97918000 | 0 | 0 | 0 |
| MPO | na | 3.06E+08 | 86389000 | 6.29E+08 | 1.44E+09 | 5.32E+09 | 5.35E+09 | 1.75E+09 | 3.21E+09 | 4.17E+09 |
| MPIG6B | na | 98892000 | 0 | 27726000 | 41482000 | 14046000 | 30413000 | 24696000 | 26795000 | 15824000 |
| MMRN1 | na | 3.73E+09 | 3.08E+08 | 1.03E+09 | 7.08E+08 | 4.87E+08 | 5.25E+08 | 8.17E+08 | 6.02E+08 | 3.51E+08 |
| MMP9 | na | 0 | 0 | 0 | 4856200 | 0 | 9618400 | 0 | 3714700 | 6408600 |
| MMP16 | na | 7.37E+08 | 7.06E+08 | 3.07E+08 | 4.04E+08 | 8.18E+08 | 5.68E+08 | 3.77E+08 | 2.64E+08 | 6.29E+08 |
| MME | na | 0 | 0 | 1.35E+08 | 3968800 | 0 | 0 | 11280000 | 0 | 7951500 |
| MIA3 | na | 0 | 0 | 0 | 0 | 0 | 0 | 0 | 0 | 0 |
| MFGE8 | na | 13842000 | 14925000 | 39598000 | 18801000 | 25867000 | 28957000 | 0 | 29746000 | 16388000 |
| MBL2 | na | 4.94E+08 | 53973000 | 2.1E+08 | 1.19E+09 | 3.02E+09 | 1.19E+09 | 1.53E+08 | 4.45E+08 | 1.23E+09 |
| MASP2 | na | 4.43E+08 | 89462000 | 5.13E+08 | 4.39E+08 | 4.23E+08 | 4.76E+08 | 5.67E+08 | 4.25E+08 | 4.46E+08 |
| MASP1 | na | 1.91E+09 | 2.98E+08 | 2.08E+09 | 1.59E+09 | 1.63E+09 | 1.77E+09 | 2.31E+09 | 1.15E+09 | 1.45E+09 |
| MAPRE2 | na | 6889900 | 0 | 5561800 | 3732600 | 6296000 | 10985000 | 0 | 4540500 | 0 |
| MAN1A1 | na | 0 | 0 | 0 | 0 | 0 | 3384800 | 0 | 5733800 | 0 |
| LYZ | na | 61719000 | 47678000 | 78908000 | 47686000 | 75271000 | 64800000 | 58328000 | 46397000 | 46603000 |
| LUM | na | 1.77E+08 | 2.48E+08 | 33533000 | 57839000 | 68670000 | 60308000 | 49547000 | 56102000 | 0 |
| LTF | na | 3.7E+08 | 56345000 | 4.28E+08 | 9.68E+08 | 2.64E+09 | 3E+09 | 7.32E+08 | 1.59E+09 | 2.44E+09 |
| LTBP1 | na | 19845000 | 16041000 | 10009000 | 12728000 | 0 | 0 | 11224000 | 9190500 | 6309200 |
| LRP1 | na | 6.21E+09 | 1.21E+09 | 1.41E+10 | 7.37E+09 | 6.88E+09 | 4.78E+09 | 7.03E+09 | 4.65E+09 | 8.11E+09 |
| LRG1 | na | 7506800 | 5.69E+08 | 21663000 | 52441000 | 81477000 | 51681000 | 48261000 | 1.84E+08 | 9761500 |
| LPA | na | 1.51E+09 | 2.8E+08 | 1.52E+10 | 4.31E+09 | 5.66E+09 | 3.48E+09 | 1.46E+09 | 3.29E+09 | 1.13E+09 |
| LMNB1 | na | 3.55E+08 | 0 | 3.22E+08 | 5250800 | 96646000 | 41559000 | 45062000 | 33323000 | 0 |
| LIMS1 | na | 2.41E+08 | 45439000 | 1.67E+08 | 1.23E+08 | 99368000 | 1.09E+08 | 76757000 | 38833000 | 30788000 |
| LGALSL | na | 3254300 | 0 | 0 | 0 | 0 | 0 | 0 | 0 | 0 |
| LGALS3BP | na | 2.26E+10 | 4.52E+09 | 2.81E+10 | 2.96E+10 | 1.38E+10 | 9.53E+09 | 1.54E+10 | 2.22E+10 | 4.46E+10 |
| LDHB | na | 42408000 | 0 | 23742000 | 20784000 | 14780000 | 0 | 0 | 2.42E+08 | 0 |
| LDHA | na | 9987300 | 0 | 0 | 7436700 | 0 | 8249400 | 0 | 12382000 | 0 |
| LCN2 | na | 0 | 0 | 14831000 | 19721000 | 22591000 | 13955000 | 0 | 9911800 | 34273000 |
| LCAT | na | 0 | 1.32E+08 | 0 | 0 | 0 | 22994000 | 15492000 | 38554000 | 0 |
| LBR | na | 0 | 0 | 0 | 0 | 0 | 9720300 | 0 | 2123400 | 0 |
| LBP | na | 7.18E+08 | 1.79E+08 | 6.21E+08 | 2.39E+08 | 4.93E+08 | 2.28E+08 | 1.87E+08 | 7.14E+08 | 5.64E+08 |
| LAMP2 | na | 0 | 18625000 | 0 | 13841000 | 22126000 | 20367000 | 11008000 | 12975000 | 18460000 |
| KRT6B | na | 73892000 | 0 | 4.21E+08 | 92779000 | 2.32E+08 | 86183000 | 0 | 1.08E+08 | 0 |
| KRT2 | na | 1.74E+09 | 6.25E+08 | 3.39E+09 | 1.17E+09 | 2.15E+09 | 1.15E+09 | 5.91E+08 | 1.16E+09 | 6.68E+08 |
| KPRP | na | 0 | 0 | 25277000 | 0 | 17504000 | 14340000 | 0 | 0 | 0 |
| KNG1 | na | 8.58E+08 | 8.63E+09 | 1.64E+09 | 1.78E+09 | 2.1E+09 | 1.83E+09 | 2.05E+09 | 2.44E+09 | 9.42E+08 |
| KLKB1 | na | 1.27E+09 | 1.65E+09 | 2.42E+09 | 1.92E+09 | 1.6E+09 | 1.39E+09 | 1.78E+09 | 1.3E+09 | 1.39E+09 |
| KIAA1958 | na | 15018000 | 16140000 | 15751000 | 30421000 | 0 | 35445000 | 37702000 | 0 | 26817000 |
| KCTD12 | na | 1.47E+08 | 0 | 69169000 | 17174000 | 7737400 | 0 | 1.1E+08 | 27204000 | 1.46E+08 |
| JUP | na | 2776700 | 0 | 6173700 | 6292600 | 4838000 | 6426000 | 0 | 2854000 | 0 |
| JCHAIN | na | 1.31E+11 | 1.11E+10 | 6.51E+10 | 3.82E+10 | 3.43E+10 | 3.55E+10 | 7.12E+10 | 2.98E+10 | 1.28E+11 |
| ITLN1 | na | 18237000 | 16075000 | 27691000 | 15152000 | 42878000 | 13194000 | 42619000 | 3154500 | 25189000 |
| ITIH4 | na | 2.74E+10 | 1.77E+10 | 5.5E+10 | 4.11E+10 | 3.65E+10 | 2.99E+10 | 3.43E+10 | 3.71E+10 | 4.21E+10 |
| ITIH3 | na | 23548000 | 1.15E+09 | 65783000 | 3.22E+08 | 3.01E+08 | 3.05E+08 | 1.68E+08 | 2.45E+08 | 1.39E+08 |
| ITIH2 | na | 9.39E+08 | 1.04E+10 | 1.61E+09 | 3.58E+09 | 2.71E+09 | 4.36E+09 | 3.59E+09 | 4.48E+09 | 1.55E+09 |
| ITIH1 | na | 8.06E+08 | 7.69E+09 | 1.49E+09 | 3.12E+09 | 2.3E+09 | 3.72E+09 | 2.83E+09 | 3.6E+09 | 8.31E+08 |
| ITGB6 | na | 5.4E+08 | 13064000 | 2.03E+08 | 91377000 | 1.36E+08 | 63371000 | 81937000 | 55070000 | 59916000 |
| ITGB3 | na | 4.76E+09 | 5.97E+08 | 2.44E+09 | 2.31E+09 | 2.73E+09 | 2.57E+09 | 1.68E+09 | 1.37E+09 | 1.29E+09 |
| ITGB2 | na | 20701000 | 0 | 16706000 | 10266000 | 24754000 | 11922000 | 12726000 | 13807000 | 34660000 |
| ITGB1 | na | 3.81E+08 | 27541000 | 2.68E+08 | 1.44E+08 | 3.16E+08 | 1.55E+08 | 92959000 | 54076000 | 97308000 |
| ITGAM | na | 0 | 0 | 4020100 | 5009500 | 53445000 | 27574000 | 9667000 | 13484000 | 79222000 |
| ITGA6 | na | 1.27E+09 | 6815400 | 5.32E+08 | 2.63E+08 | 6.55E+08 | 3.09E+08 | 2.07E+08 | 75258000 | 25230000 |
| ITGA2B | na | 7.54E+09 | 6.11E+08 | 3.24E+09 | 3.48E+09 | 3.99E+09 | 3.72E+09 | 2.77E+09 | 2.09E+09 | 1.73E+09 |
| ITGA2 | na | 22592000 | 0 | 0 | 10442000 | 0 | 0 | 0 | 0 | 0 |
| IMMT | na | 1.25E+08 | 0 | 0 | 0 | 0 | 0 | 0 | 0 | 0 |
| ILK | na | 2.6E+08 | 0 | 47778000 | 44679000 | 44621000 | 42559000 | 54430000 | 21114000 | 16639000 |
| IGSF8 | na | 0 | 0 | 0 | 0 | 10941000 | 0 | 0 | 0 | 0 |
| IGLV9-49 | na | 0 | 0 | 4.26E+08 | 70164000 | 0 | 71704000 | 1.26E+08 | 92008000 | 0 |
| IGLV8-61 | na | 1.82E+09 | 7.74E+08 | 2.96E+09 | 2.43E+09 | 8.75E+08 | 3.57E+09 | 1.94E+09 | 3.78E+09 | 3.81E+09 |
| IGLV7-46 | na | 2.98E+09 | 5.11E+08 | 1.63E+09 | 7.83E+08 | 5.23E+08 | 8.66E+08 | 1.5E+09 | 8.26E+08 | 6.33E+09 |
| IGLV6-57 | na | 2.95E+08 | 82149000 | 3.38E+08 | 1.34E+08 | 50242000 | 62848000 | 71614000 | 96829000 | 1.66E+08 |
| IGLV5-45 | na | 2.65E+09 | 1.29E+08 | 2.9E+09 | 4.44E+08 | 0 | 4.83E+08 | 7.7E+08 | 4.17E+08 | 9.81E+08 |
| IGLV5-39 | na | 7.13E+08 | 23112000 | 3.76E+08 | 0 | 0 | 75730000 | 68610000 | 58020000 | 90131000 |
| IGLV5-37 | na | 0 | 0 | 0 | 0 | 0 | 9126600 | 0 | 0 | 45480000 |
| IGLV4-69 | na | 7.32E+08 | 1.15E+08 | 1.3E+09 | 1.17E+09 | 95127000 | 4.78E+08 | 3.93E+08 | 2.38E+08 | 4.58E+08 |
| IGLV4-60 | na | 0 | 1.78E+08 | 1.65E+08 | 2.56E+08 | 84772000 | 1.02E+08 | 38099000 | 46966000 | 0 |
| IGLV4-3 | na | 3.69E+09 | 0 | 1.46E+09 | 1.98E+08 | 2.67E+08 | 2.58E+08 | 1.52E+08 | 3.48E+08 | 6.37E+08 |
| IGLV3-9 | na | 1.07E+09 | 1.25E+09 | 6.67E+08 | 1.62E+09 | 6.21E+08 | 8.65E+08 | 5.52E+08 | 1.74E+09 | 7.72E+08 |
| IGLV3-27 | na | 1.04E+08 | 37045000 | 1.66E+08 | 2.43E+08 | 89866000 | 1.79E+08 | 97652000 | 1.72E+08 | 2.5E+08 |
| IGLV3-25 | na | 2.31E+08 | 1.81E+08 | 3.47E+08 | 4.64E+08 | 95853000 | 2.61E+08 | 2.2E+08 | 1.44E+08 | 4.61E+08 |
| IGLV3-21 | na | 88305000 | 2.29E+08 | 2.8E+08 | 79825000 | 88983000 | 2E+08 | 1.58E+08 | 1.35E+08 | 2.25E+08 |
| IGLV3-19 | na | 2.51E+09 | 8.12E+08 | 2.42E+09 | 1.14E+09 | 7.39E+08 | 1.22E+09 | 1.23E+09 | 3.2E+09 | 1.9E+09 |
| IGLV3-16 | na | 2.05E+09 | 3.73E+08 | 2.32E+09 | 2.06E+09 | 9.9E+08 | 2.37E+09 | 1.24E+09 | 9.41E+08 | 3.26E+09 |
| IGLV3-10 | na | 2.19E+09 | 5.03E+08 | 1.49E+09 | 1.11E+09 | 1.49E+09 | 6.03E+08 | 6.59E+08 | 2.66E+09 | 1.88E+09 |
| IGLV3-1 | na | 94989000 | 97245000 | 2.72E+08 | 1.17E+08 | 56548000 | 67256000 | 1.38E+08 | 6.09E+08 | 1.01E+08 |
| IGLV2-8 | na | 7.27E+09 | 2.09E+08 | 3.24E+09 | 1.82E+09 | 1.23E+09 | 2E+09 | 2.09E+09 | 1.59E+09 | 2.29E+09 |
| IGLV2-23 | na | 6.25E+08 | 1.2E+08 | 0 | 3.47E+08 | 0 | 0 | 1.82E+08 | 5.4E+08 | 6.03E+08 |
| IGLV2-18 | na | 2.91E+09 | 3.56E+08 | 2.57E+09 | 1.77E+08 | 2.25E+08 | 7.35E+08 | 1.98E+09 | 2.4E+08 | 1.12E+09 |
| IGLV2-14 | na | 2.81E+08 | 2.17E+08 | 1.67E+09 | 1.05E+09 | 5.52E+08 | 4.57E+08 | 2.47E+08 | 8.35E+08 | 1.35E+09 |
| IGLV2-11 | na | 3.98E+09 | 7.58E+08 | 2.56E+09 | 8.89E+08 | 4.44E+08 | 1.56E+09 | 2.71E+09 | 1.34E+09 | 2.97E+09 |
| IGLV1-51 | na | 4.27E+09 | 9.8E+08 | 2.11E+09 | 6.89E+08 | 1.15E+09 | 1.55E+09 | 2.26E+09 | 1.72E+09 | 3.42E+09 |
| IGLV1-47 | na | 4.1E+09 | 1.99E+09 | 4.34E+09 | 1.94E+09 | 1.84E+09 | 2.07E+09 | 3.53E+09 | 3.37E+09 | 3.95E+09 |
| IGLV1-44 | na | 3.17E+08 | 57174000 | 1.61E+08 | 47554000 | 26455000 | 64084000 | 74909000 | 1.63E+08 | 89103000 |
| IGLV1-40 | na | 1.21E+09 | 7.36E+08 | 2.8E+09 | 8.2E+08 | 5.11E+08 | 1.53E+09 | 1.59E+09 | 2.21E+09 | 2.51E+09 |
| IGLV1-36 | na | 26819000 | 29507000 | 35964000 | 5407900 | 15306000 | 7371100 | 24048000 | 11183000 | 34722000 |
| IGLV10-54 | na | 5.85E+08 | 15882000 | 3.65E+08 | 71569000 | 12365000 | 31928000 | 0 | 2.73E+08 | 9.21E+08 |
| IGLL5 | na | 1.79E+11 | 7.36E+10 | 9.36E+10 | 6.85E+10 | 6.24E+10 | 7.51E+10 | 1.18E+11 | 7.44E+10 | 1.59E+11 |
| IGLL1 | na | 33245000 | 54390000 | 26112000 | 28142000 | 25899000 | 21251000 | 10089000 | 42145000 | 35182000 |
| IGLC7 | na | 75956000 | 4668000 | 31681000 | 8998700 | 36979000 | 24552000 | 35884000 | 9973600 | 47750000 |
| IGLC3 | na | 2.94E+10 | 7.14E+09 | 1.73E+10 | 8.23E+09 | 1.04E+10 | 1.3E+10 | 1.29E+10 | 1.49E+10 | 2.49E+10 |
| IGKV6D-21 | na | 9.34E+08 | 3.06E+08 | 1.01E+09 | 2.06E+08 | 2.46E+08 | 2.19E+08 | 4.76E+08 | 3.51E+08 | 8.11E+08 |
| IGKV6-21 | na | 1.36E+08 | 1.37E+08 | 1.43E+08 | 27940000 | 51311000 | 1.81E+08 | 28000000 | 58558000 | 1.35E+08 |
| IGKV4-1 | na | 1.57E+10 | 2.2E+09 | 1.16E+10 | 4.45E+09 | 3.08E+09 | 4.73E+09 | 1.85E+10 | 7.01E+09 | 2.29E+10 |
| IGKV3D-7 | na | 17478000 | 0 | 18856000 | 0 | 0 | 12856000 | 48156000 | 0 | 0 |
| IGKV3D-20 | na | 1.35E+09 | 5.4E+08 | 7.78E+08 | 1.07E+08 | 9.68E+08 | 7.69E+08 | 1.11E+09 | 5.72E+08 | 9.49E+08 |
| IGKV3D-15 | na | 22649000 | 5.71E+08 | 0 | 5.29E+08 | 1.85E+08 | 2.42E+08 | 4.32E+08 | 2E+08 | 3.12E+08 |
| IGKV3D-11 | na | 9.34E+09 | 2.34E+09 | 9.3E+09 | 4.18E+09 | 3.66E+09 | 3.54E+09 | 6.62E+09 | 2.94E+09 | 1.27E+10 |
| IGKV3-20 | na | 2.26E+10 | 4.4E+09 | 1.46E+10 | 6.33E+09 | 3.96E+09 | 9.01E+09 | 9.56E+09 | 4.81E+09 | 1.42E+10 |
| IGKV3-15 | na | 9.66E+09 | 3.03E+09 | 4.89E+09 | 3.54E+09 | 2.13E+09 | 2.96E+09 | 3.07E+09 | 2.52E+09 | 7.07E+09 |
| IGKV2D-29 | na | 82746000 | 0 | 11425000 | 0 | 0 | 13294000 | 12936000 | 10189000 | 37528000 |
| IGKV2D-28 | na | 1.11E+09 | 54527000 | 2.25E+08 | 1.79E+08 | 72204000 | 1.9E+08 | 4.66E+08 | 3.03E+08 | 1.1E+09 |
| IGKV2-40 | na | 31107000 | 21771000 | 9236300 | 13296000 | 9594700 | 28433000 | 32412000 | 19218000 | 19469000 |
| IGKV2-30 | na | 2.38E+09 | 3.29E+08 | 5.63E+08 | 0 | 1.14E+08 | 2.4E+08 | 82075000 | 2.98E+08 | 6.28E+08 |
| IGKV2-29 | na | 3.19E+10 | 3.85E+09 | 2.13E+10 | 9.75E+09 | 9.4E+09 | 1.39E+10 | 3.47E+10 | 1.13E+10 | 3.15E+10 |
| IGKV2-24 | na | 6.62E+09 | 2.25E+09 | 3.26E+09 | 2.57E+09 | 6.45E+08 | 1.68E+09 | 6.1E+09 | 1.17E+09 | 8.41E+09 |
| IGKV1D-8 | na | 31722000 | 13923000 | 44371000 | 14168000 | 13654000 | 13203000 | 30288000 | 4212300 | 40858000 |
| IGKV1D-39 | na | 1.57E+09 | 5.63E+08 | 1.82E+09 | 6.6E+08 | 4.73E+08 | 5.41E+08 | 7.81E+08 | 5.45E+08 | 1.17E+09 |
| IGKV1D-16 | na | 2E+08 | 1.71E+08 | 1.43E+08 | 48659000 | 4.09E+08 | 42083000 | 1.04E+08 | 85350000 | 2.72E+08 |
| IGKV1D-13 | na | 3.55E+08 | 2.85E+08 | 47601000 | 2.28E+08 | 38560000 | 31522000 | 23650000 | 17979000 | 17980000 |
| IGKV1-8 | na | 7.76E+08 | 2.84E+08 | 6.22E+08 | 7.4E+08 | 3.56E+08 | 3.41E+08 | 1.39E+09 | 1.98E+08 | 8.66E+08 |
| IGKV1-5 | na | 8.75E+08 | 3.84E+08 | 6.48E+08 | 3.3E+08 | 1.81E+08 | 1.83E+08 | 7.71E+08 | 3.96E+08 | 1.36E+09 |
| IGKV1-33 | na | 1.96E+09 | 8.75E+08 | 4.41E+08 | 4.74E+08 | 3.03E+08 | 4.14E+08 | 3.38E+08 | 2.9E+08 | 5.94E+08 |
| IGKV1-27 | na | 1.81E+09 | 6.02E+08 | 9.29E+08 | 5.82E+08 | 4.2E+08 | 4.87E+08 | 9.8E+08 | 5.96E+08 | 9.76E+08 |
| IGKV1-17 | na | 3.47E+09 | 1.44E+09 | 3.16E+09 | 2.04E+09 | 7.6E+08 | 1.41E+09 | 2.14E+09 | 9.15E+08 | 2.44E+09 |
| IGKV1-16 | na | 1.56E+09 | 3.6E+08 | 9.06E+08 | 3.97E+08 | 2.5E+08 | 2.83E+08 | 7.86E+08 | 4.47E+08 | 1.61E+09 |
| IGKV1-12 | na | 2.51E+08 | 55998000 | 1.56E+08 | 1.41E+08 | 1.21E+08 | 67453000 | 1.65E+08 | 66848000 | 3.11E+08 |
| IGKC | na | 1.4E+10 | 2.02E+10 | 1.25E+10 | 8.11E+09 | 4.6E+09 | 6.99E+09 | 1E+10 | 5.92E+09 | 1.37E+10 |
| IGHV7-4-1 | na | 2.13E+08 | 86433000 | 3.85E+08 | 3.41E+08 | 0 | 2.52E+08 | 1.19E+08 | 91310000 | 1.95E+08 |
| IGHV6-1 | na | 3.91E+09 | 1.26E+09 | 1.5E+09 | 2.34E+09 | 2.75E+09 | 8.58E+08 | 3.45E+09 | 9.57E+08 | 2.52E+09 |
| IGHV5-51 | na | 6.67E+09 | 2.46E+09 | 2.56E+09 | 2.09E+09 | 1.51E+09 | 1.15E+09 | 2.4E+09 | 2.48E+09 | 4.1E+09 |
| IGHV5-10-1 | na | 40363000 | 7.39E+08 | 0 | 0 | 0 | 45464000 | 0 | 21284000 | 1.12E+09 |
| IGHV4-61 | na | 9901300 | 6278300 | 0 | 5437900 | 0 | 0 | 0 | 6353700 | 9191700 |
| IGHV4-4 | na | 1.99E+09 | 1.85E+09 | 1.93E+08 | 3.33E+08 | 1.05E+09 | 2.17E+08 | 2.21E+08 | 1.5E+08 | 1.87E+09 |
| IGHV4-39 | na | 87060000 | 19359000 | 12195000 | 12468000 | 5788000 | 22401000 | 30853000 | 28041000 | 42277000 |
| IGHV4-38-2 | na | 10903000 | 0 | 4253100 | 4316000 | 5736400 | 23370000 | 63215000 | 4264100 | 5361300 |
| IGHV4-34 | na | 6.73E+09 | 3.15E+09 | 2.99E+09 | 2.84E+09 | 1.93E+09 | 2.81E+09 | 3.24E+09 | 2.62E+09 | 3.79E+09 |
| IGHV4-30-2 | na | 2.22E+08 | 2.88E+08 | 0 | 2.25E+08 | 4.98E+08 | 4.56E+08 | 2.47E+08 | 2.19E+08 | 1.51E+08 |
| IGHV4-28 | na | 3.87E+09 | 1.39E+09 | 1.32E+09 | 5.95E+08 | 2.27E+09 | 8.84E+08 | 1.27E+09 | 3.37E+08 | 2.75E+09 |
| IGHV3-9 | na | 5.04E+09 | 8.78E+08 | 2.28E+09 | 1.33E+09 | 9.97E+08 | 2.07E+09 | 1.96E+09 | 1.17E+09 | 4.41E+08 |
| IGHV3-74 | na | 1.2E+10 | 1.14E+09 | 7.35E+09 | 2.03E+09 | 1.59E+09 | 1.89E+09 | 4.59E+09 | 4.17E+09 | 6.48E+09 |
| IGHV3-73 | na | 2.5E+09 | 6.47E+08 | 1.29E+09 | 8.64E+08 | 2.12E+08 | 6.41E+08 | 9.23E+08 | 6.97E+08 | 1.89E+09 |
| IGHV3-72 | na | 4.64E+09 | 1.19E+09 | 3.25E+09 | 1E+09 | 6.57E+08 | 1.65E+09 | 2.41E+09 | 8.06E+08 | 3.66E+09 |
| IGHV3-7 | na | 1.47E+10 | 2.27E+09 | 7.81E+09 | 4.35E+09 | 2.98E+09 | 4.38E+09 | 7.93E+09 | 3.62E+09 | 9.58E+09 |
| IGHV3-66 | na | 1.6E+08 | 8095600 | 83683000 | 21059000 | 14652000 | 34548000 | 26159000 | 14499000 | 1.45E+08 |
| IGHV3-64D | na | 1.04E+09 | 5.85E+08 | 3.06E+08 | 2.61E+08 | 64974000 | 3.66E+08 | 4.88E+08 | 2.71E+08 | 1.47E+09 |
| IGHV3-64 | na | 5.13E+08 | 1.35E+08 | 1.08E+08 | 1.1E+08 | 38495000 | 1.13E+08 | 1.41E+08 | 39015000 | 3.12E+08 |
| IGHV3-49 | na | 1.61E+09 | 1.71E+09 | 9.33E+08 | 4.43E+08 | 4.2E+08 | 1.11E+09 | 1.14E+09 | 9.96E+08 | 1.65E+09 |
| IGHV3-48 | na | 8.33E+09 | 2.11E+09 | 3.23E+09 | 3.42E+09 | 1.26E+09 | 2.38E+09 | 6.76E+09 | 3.71E+09 | 4.81E+09 |
| IGHV3-43D | na | 0 | 0 | 0 | 0 | 0 | 0 | 0 | 0 | 0 |
| IGHV3-43 | na | 5.75E+08 | 2.78E+08 | 2.65E+08 | 68648000 | 28409000 | 0 | 1.45E+08 | 3.12E+08 | 2.89E+08 |
| IGHV3-33 | na | 9.01E+09 | 2.62E+09 | 4.32E+09 | 3.86E+09 | 1.75E+09 | 2.96E+09 | 3.92E+09 | 2.86E+09 | 6.48E+09 |
| IGHV3-30-5 | na | 1.47E+10 | 2.73E+09 | 9.14E+09 | 7.41E+09 | 4.61E+09 | 5.42E+09 | 8.8E+09 | 3.63E+09 | 9.66E+09 |
| IGHV3-30-3 | na | 1.21E+08 | 22474000 | 79600000 | 34827000 | 39393000 | 38775000 | 47120000 | 79957000 | 85666000 |
| IGHV3-23 | na | 1.16E+08 | 48657000 | 95478000 | 77031000 | 32365000 | 46573000 | 67838000 | 29967000 | 55068000 |
| IGHV3-21 | na | 93346000 | 5491100 | 51703000 | 20064000 | 20799000 | 8791900 | 35119000 | 33052000 | 80811000 |
| IGHV3-20 | na | 1.79E+08 | 0 | 1.02E+09 | 87075000 | 74563000 | 3.29E+08 | 55631000 | 27946000 | 2.21E+08 |
| IGHV3-15 | na | 1.62E+10 | 2.69E+09 | 7.69E+09 | 3.16E+09 | 2.13E+09 | 3.52E+09 | 3.32E+09 | 3.95E+09 | 9.79E+09 |
| IGHV3-13 | na | 2.88E+09 | 1.29E+08 | 1.07E+09 | 2.9E+08 | 1.69E+08 | 4.29E+08 | 6.48E+08 | 2.96E+08 | 8.97E+08 |
| IGHV3-11 | na | 38767000 | 0 | 15468000 | 16784000 | 0 | 19373000 | 17879000 | 14454000 | 56946000 |
| IGHV2-70D | na | 29909000 | 1.35E+08 | 1.1E+08 | 0 | 36818000 | 92001000 | 0 | 19210000 | 1.77E+08 |
| IGHV2-70 | na | 0 | 0 | 0 | 25242000 | 0 | 0 | 0 | 0 | 53017000 |
| IGHV2-5 | na | 2.73E+08 | 7122500 | 1.77E+08 | 91621000 | 54068000 | 1.24E+08 | 2.17E+08 | 14312000 | 3.74E+08 |
| IGHV2-26 | na | 1.59E+09 | 1.49E+08 | 1.88E+08 | 2.52E+08 | 90848000 | 1.18E+08 | 0 | 2.33E+08 | 3.11E+08 |
| IGHV1-8 | na | 3.66E+08 | 0 | 1.32E+08 | 69386000 | 23549000 | 34877000 | 81687000 | 1.14E+08 | 59667000 |
| IGHV1-69-2 | na | 5.68E+09 | 4.01E+09 | 2.78E+09 | 3.87E+09 | 1.93E+09 | 3.01E+09 | 2.46E+09 | 2.84E+09 | 6.54E+09 |
| IGHV1-58 | na | 40195000 | 28967000 | 21570000 | 7223900 | 16359000 | 9789200 | 0 | 14036000 | 17725000 |
| IGHV1-46 | na | 6.86E+08 | 36623000 | 1.4E+08 | 1.56E+08 | 48911000 | 92771000 | 89486000 | 1.16E+08 | 2.22E+08 |
| IGHV1-45 | na | 3.49E+08 | 95610000 | 86158000 | 2.65E+08 | 1.12E+08 | 1.57E+08 | 1.24E+08 | 62194000 | 3.77E+08 |
| IGHV1-3 | na | 4.17E+09 | 4.77E+08 | 5.39E+08 | 7.54E+08 | 1.08E+08 | 5.18E+08 | 2.82E+08 | 3.57E+08 | 1.39E+09 |
| IGHV1-24 | na | 51574000 | 93617000 | 0 | 24178000 | 0 | 23668000 | 33953000 | 21896000 | 55866000 |
| IGHV1-2 | na | 3.76E+09 | 8.73E+08 | 1.55E+09 | 1.46E+09 | 1.07E+09 | 9.68E+08 | 1.78E+09 | 2.74E+09 | 1.62E+09 |
| IGHV1-18 | na | 6.39E+08 | 3.42E+08 | 1.65E+08 | 80081000 | 76512000 | 82096000 | 64947000 | 2.94E+08 | 1.82E+08 |
| IGHM | na | 1.31E+12 | 2.18E+11 | 9.70E+11 | 5.09E+11 | 3.66E+11 | 4.38E+11 | 9.38E+11 | 4.14E+11 | 1.41E+12 |
| IGHG4 | na | 8.86E+08 | 4.07E+09 | 2.02E+09 | 6.03E+09 | 4.79E+09 | 3.49E+09 | 9.34E+09 | 2.84E+09 | 3.87E+08 |
| IGHG3 | na | 9.22E+09 | 1.64E+10 | 1.08E+10 | 1.54E+10 | 1.37E+10 | 1.04E+10 | 3.68E+09 | 6.05E+09 | 1.3E+10 |
| IGHG2 | na | 1.84E+10 | 7.78E+10 | 3.84E+10 | 7.69E+10 | 6.01E+10 | 7.82E+10 | 3.81E+10 | 3.23E+10 | 1.81E+10 |
| IGHG1 | na | 4.58E+10 | 4.33E+11 | 5.72E+10 | 1.24E+11 | 1.19E+11 | 1.31E+11 | 1.38E+11 | 1.35E+11 | 9.86E+10 |
| IGHE | na | 2.47E+08 | 1.66E+08 | 64931000 | 1.41E+08 | 1.59E+08 | 1.25E+08 | 2.31E+08 | 1.07E+08 | 3.53E+08 |
| IGHD | na | 4.57E+08 | 2.58E+09 | 1.93E+08 | 1.84E+08 | 7.7E+08 | 1.2E+09 | 9.76E+08 | 3.5E+08 | 6.56E+08 |
| IGHA2 | na | 8301200 | 12963000 | 0 | 20794000 | 28964000 | 19532000 | 0 | 0 | 0 |
| IGHA1 | na | 5.31E+10 | 8.48E+10 | 4.75E+10 | 5.68E+10 | 5.82E+10 | 1.01E+11 | 4.27E+10 | 8.44E+10 | 3.15E+10 |
| IGFBP3 | na | 0 | 34702000 | 0 | 13938000 | 0 | 7775300 | 0 | 0 | 0 |
| IGFALS | na | 4401100 | 1.05E+09 | 48082000 | 4.62E+08 | 1.22E+08 | 1.46E+08 | 1.05E+08 | 2.59E+08 | 49513000 |
| IGF2 | na | 0 | 25532000 | 0 | 0 | 0 | 0 | 0 | 0 | 0 |
| IFITM1 | na | 44240000 | 0 | 21902000 | 29509000 | 84622000 | 28905000 | 31752000 | 0 | 45835000 |
| IDE | na | 0 | 5.01E+08 | 1.65E+08 | 51521000 | 1.68E+08 | 2.1E+08 | 38772000 | 24603000 | 3.27E+08 |
| ICAM1 | na | 0 | 0 | 0 | 0 | 29699000 | 22933000 | 0 | 15378000 | 74631000 |
| HYDIN | na | 1.46E+08 | 2.48E+08 | 2.5E+08 | 4.34E+08 | 3.41E+08 | 4.06E+08 | 2.48E+08 | 3.93E+08 | 2.02E+08 |
| HSPA8 | na | 2.84E+08 | 5078900 | 1.58E+08 | 64686000 | 1.31E+08 | 1.72E+08 | 12941000 | 1.66E+08 | 15301000 |
| HSPA6 | na | 1.75E+08 | 0 | 58111000 | 41287000 | 83469000 | 61300000 | 0 | 32053000 | 91663000 |
| HSPA5 | na | 13305000 | 11391000 | 9244800 | 20557000 | 20477000 | 12473000 | 0 | 8746900 | 11953000 |
| HSPA1B | na | 0 | 0 | 0 | 0 | 0 | 0 | 0 | 9906800 | 0 |
| HSP90B1 | na | 0 | 0 | 0 | 0 | 4677400 | 3264400 | 0 | 3894900 | 4084600 |
| HSP90AB1 | na | 0 | 0 | 0 | 0 | 0 | 0 | 0 | 11502000 | 0 |
| HSP90AA1 | na | 19952000 | 0 | 0 | 10916000 | 0 | 17794000 | 0 | 23874000 | 0 |
| HRG | na | 1.8E+09 | 8.35E+09 | 2.03E+09 | 2.54E+09 | 3.05E+09 | 2.76E+09 | 3.71E+09 | 2.27E+09 | 1.66E+09 |
| HPX | na | 1.08E+09 | 3.63E+10 | 2.94E+09 | 5.43E+09 | 6.19E+09 | 8.66E+09 | 6.84E+09 | 1.1E+10 | 9.85E+08 |
| HPR | na | 2.06E+10 | 3.31E+09 | 1.41E+10 | 1.45E+10 | 6.22E+09 | 5.81E+09 | 1.87E+10 | 7.21E+09 | 2.79E+10 |
| HP | na | 4.65E+10 | 1.06E+11 | 4.55E+10 | 7.89E+10 | 6.07E+10 | 4.04E+10 | 6.28E+10 | 3.23E+11 | 1.11E+11 |
| HLA-G | na | 7808000 | 0 | 0 | 12971000 | 0 | 0 | 0 | 0 | 0 |
| HLA-DRA | na | 10371000 | 0 | 8883100 | 6124800 | 8611600 | 7689100 | 5951000 | 4272700 | 0 |
| HLA-C | na | 0 | 14306000 | 0 | 0 | 0 | 0 | 0 | 0 | 0 |
| HLA-B | na | 1.96E+08 | 0 | 55613000 | 0 | 0 | 0 | 33637000 | 0 | 0 |
| HLA-A | na | 1.87E+08 | 26250000 | 79482000 | 87661000 | 87986000 | 76627000 | 92567000 | 1.01E+08 | 55091000 |
| HIST1H4A | na | 1.5E+08 | 86143000 | 3.69E+08 | 1.89E+09 | 5.23E+09 | 4.06E+09 | 8.09E+08 | 3.33E+09 | 6.18E+09 |
| HIST1H3A | na | 22646000 | 19092000 | 1.11E+08 | 5.6E+08 | 2.25E+09 | 2.11E+09 | 3.34E+08 | 2.22E+09 | 2.48E+09 |
| HIST1H2BL | na | 21587000 | 17615000 | 1.12E+08 | 9.39E+08 | 2.52E+09 | 1.84E+09 | 3.78E+08 | 1.79E+09 | 2.58E+09 |
| HIST1H2AJ | na | 27169000 | 0 | 1.24E+08 | 6.64E+08 | 1.88E+09 | 1.9E+09 | 3.53E+08 | 1.29E+09 | 2.44E+09 |
| HEG1 | na | 0 | 0 | 0 | 0 | 13437000 | 13855000 | 0 | 0 | 0 |
| HBG2 | na | 0 | 0 | 0 | 33204000 | 7951500 | 15640000 | 0 | 21402000 | 6391000 |
| HBD | na | 0 | 24078000 | 0 | 3.13E+08 | 3.74E+08 | 2.58E+08 | 0 | 5.38E+08 | 12950000 |
| HBB | na | 5.22E+09 | 6.38E+09 | 1.07E+10 | 3.77E+10 | 3.52E+10 | 2.8E+10 | 5.74E+09 | 5.35E+10 | 1.74E+10 |
| HBA1 | na | 2.25E+09 | 6.22E+09 | 5.66E+09 | 2.19E+10 | 1.19E+10 | 1.23E+10 | 3E+09 | 2.59E+10 | 6.59E+09 |
| HABP2 | na | 0 | 4.2E+08 | 14122000 | 1.16E+08 | 1.45E+08 | 93485000 | 20841000 | 2.23E+08 | 15901000 |
| GYPA | na | 0 | 0 | 0 | 11784000 | 11283000 | 0 | 0 | 8606900 | 0 |
| GPX3 | na | 1.01E+08 | 2.67E+08 | 41627000 | 1.7E+08 | 1.05E+08 | 1.18E+08 | 1.64E+09 | 1.01E+08 | 34627000 |
| GPRC5C | na | 0 | 0 | 42399000 | 0 | 0 | 0 | 0 | 0 | 0 |
| GPLD1 | na | 13453000 | 3.33E+08 | 19589000 | 47116000 | 54124000 | 49376000 | 46335000 | 33976000 | 0 |
| GP9 | na | 3.15E+08 | 0 | 1.06E+08 | 1.7E+08 | 1.39E+08 | 1.37E+08 | 88479000 | 88782000 | 61260000 |
| GP1BB | na | 8.55E+08 | 0 | 3.2E+08 | 3.43E+08 | 4.6E+08 | 3.67E+08 | 2.62E+08 | 1.74E+08 | 1.56E+08 |
| GP1BA | na | 2.76E+08 | 66676000 | 72198000 | 64178000 | 1.02E+08 | 78841000 | 35055000 | 42239000 | 25779000 |
| GNB2 | na | 1.3E+08 | 0 | 94180000 | 62373000 | 75231000 | 68963000 | 0 | 90349000 | 0 |
| GNB1 | na | 36673000 | 0 | 15580000 | 7831000 | 7157700 | 16002000 | 0 | 5095900 | 0 |
| GNAQ | na | 22884000 | 0 | 13690000 | 9153600 | 0 | 5458800 | 0 | 8395500 | 0 |
| GNAI2 | na | 1.68E+08 | 43322000 | 99750000 | 1.32E+08 | 87058000 | 1.03E+08 | 56917000 | 50932000 | 97763000 |
| GLUD1 | na | 48226000 | 0 | 1.71E+09 | 0 | 0 | 0 | 0 | 0 | 0 |
| GGT3P | na | 0 | 0 | 1.31E+08 | 14060000 | 10214000 | 8786900 | 0 | 10175000 | 12546000 |
| GCA | na | 35486000 | 19949000 | 59805000 | 78271000 | 1.76E+08 | 1.41E+08 | 40736000 | 80528000 | 1.09E+08 |
| GC | na | 3.58E+08 | 1.25E+10 | 1.38E+09 | 2.18E+09 | 2.48E+09 | 2.51E+09 | 1.95E+09 | 3.02E+09 | 3.88E+08 |
| GAPDH | na | 2.8E+08 | 0 | 2.62E+08 | 2.85E+08 | 2.19E+08 | 2.51E+08 | 1.15E+08 | 2.31E+08 | 2E+08 |
| GANAB | na | 6362500 | 0 | 0 | 3872900 | 0 | 3603900 | 26931000 | 6591900 | 7186500 |
| G6PD | na | 7448700 | 0 | 0 | 4561300 | 0 | 0 | 0 | 3422300 | 0 |
| FTL | na | 0 | 0 | 4954700 | 2.61E+08 | 91235000 | 2.25E+08 | 3.5E+08 | 4.88E+08 | 1.87E+08 |
| FTH1 | na | 0 | 0 | 0 | 29563000 | 32736000 | 33577000 | 44373000 | 50239000 | 28825000 |
| FSTL1 | na | 29241000 | 0 | 49205000 | 24750000 | 38259000 | 15729000 | 28837000 | 14989000 | 33062000 |
| FN1 | na | 3.17E+10 | 4.21E+10 | 3.16E+10 | 9.48E+10 | 4.68E+10 | 5.7E+10 | 9.1E+10 | 7.96E+10 | 4.45E+10 |
| FLOT2 | na | 0 | 0 | 0 | 8138000 | 0 | 7792600 | 0 | 0 | 17279000 |
| FLOT1 | na | 0 | 0 | 0 | 8940100 | 13129000 | 22466000 | 0 | 18321000 | 23182000 |
| FLNA | na | 8.28E+09 | 31809000 | 2.66E+09 | 2.2E+09 | 2.68E+09 | 2.39E+09 | 1.64E+09 | 1.11E+09 | 8.97E+08 |
| FHOD1 | na | 0 | 0 | 0 | 0 | 0 | 0 | 0 | 0 | 0 |
| FGL1 | na | 0 | 0 | 0 | 0 | 0 | 0 | 0 | 6741000 | 0 |
| FGG | na | 1.25E+09 | 1.31E+09 | 1.76E+09 | 1.21E+09 | 1.41E+09 | 9.52E+08 | 9.52E+08 | 3.19E+09 | 1.83E+09 |
| FGB | na | 1.21E+09 | 1.16E+09 | 1.18E+09 | 9.07E+08 | 1.34E+09 | 7.97E+08 | 7.17E+08 | 2.79E+09 | 1.26E+09 |
| FGA | na | 1.47E+09 | 1.4E+09 | 1.53E+09 | 1.62E+09 | 1.56E+09 | 9.53E+08 | 1.43E+09 | 2.75E+09 | 1.72E+09 |
| FETUB | na | 13353000 | 2.29E+08 | 8053600 | 34234000 | 9313900 | 23400000 | 12172000 | 15544000 | 5952800 |
| FERMT3 | na | 2.41E+09 | 21458000 | 7.49E+08 | 5.71E+08 | 6.96E+08 | 8.37E+08 | 3.81E+08 | 3.04E+08 | 1.9E+08 |
| FCN3 | na | 1.5E+10 | 2.35E+09 | 1.6E+10 | 1.12E+10 | 5.66E+09 | 1.41E+10 | 1.93E+10 | 1.11E+10 | 1.13E+10 |
| FCN2 | na | 2.66E+09 | 4.62E+08 | 3.93E+09 | 2.04E+09 | 2.64E+09 | 6.37E+08 | 1.07E+09 | 6.62E+08 | 1.02E+09 |
| FCN1 | na | 2.18E+08 | 29128000 | 6.68E+08 | 3.15E+08 | 2.21E+08 | 68223000 | 3.86E+08 | 2.57E+08 | 4.03E+08 |
| FCMR | na | 10474000 | 0 | 0 | 3880100 | 0 | 0 | 0 | 0 | 8409400 |
| FCGR3B | na | 9933700 | 42804000 | 16263000 | 15623000 | 19041000 | 10852000 | 31320000 | 13294000 | 0 |
| FCGBP | na | 7.85E+09 | 1.67E+09 | 1.12E+10 | 3.04E+09 | 2.69E+09 | 4.39E+09 | 4.86E+09 | 2.57E+09 | 1.25E+10 |
| FBN1 | na | 0 | 0 | 21173000 | 0 | 0 | 0 | 0 | 0 | 0 |
| FBLN1 | na | 5.02E+08 | 2.51E+08 | 1.71E+08 | 91835000 | 1.45E+08 | 99828000 | 1.26E+08 | 98186000 | 81948000 |
| FAM83B | na | 0 | 0 | 1.23E+08 | 42251000 | 0 | 91740000 | 1.5E+08 | 65799000 | 0 |
| FAM21C | na | 0 | 0 | 0 | 0 | 87730000 | 0 | 3.09E+08 | 0 | 0 |
| FAM151A | na | 0 | 0 | 72043000 | 0 | 0 | 0 | 0 | 0 | 0 |
| FABP5 | na | 0 | 0 | 0 | 0 | 0 | 0 | 0 | 0 | 0 |
| F9 | na | 9.78E+08 | 7E+08 | 5E+08 | 1.68E+09 | 1.27E+09 | 1.08E+09 | 1.75E+09 | 1.01E+09 | 1.24E+09 |
| F7 | na | 0 | 31705000 | 15028000 | 23132000 | 22129000 | 24382000 | 69754000 | 23200000 | 9845600 |
| F5 | na | 3.2E+09 | 4.39E+08 | 4.78E+08 | 8.87E+08 | 5.64E+08 | 5.92E+08 | 7.34E+08 | 6.51E+08 | 3.15E+08 |
| F2 | na | 1.04E+10 | 1.7E+10 | 1.3E+10 | 1.72E+10 | 1.17E+10 | 1.58E+10 | 2.3E+10 | 1.68E+10 | 1.89E+10 |
| F13B | na | 11255000 | 2.78E+08 | 25483000 | 67479000 | 66556000 | 74955000 | 92939000 | 1.07E+08 | 0 |
| F13A1 | na | 7.03E+08 | 3.97E+08 | 2.52E+08 | 2.74E+08 | 2.95E+08 | 2.35E+08 | 4.84E+08 | 2.41E+08 | 83802000 |
| F12 | na | 26490000 | 4.21E+08 | 0 | 1.28E+08 | 1.03E+08 | 99435000 | 1.61E+08 | 1.07E+08 | 0 |
| F11R | na | 2.4E+08 | 82183000 | 49996000 | 57926000 | 67771000 | 52302000 | 24918000 | 27417000 | 63011000 |
| F11 | na | 74507000 | 78832000 | 1.15E+08 | 53569000 | 1.15E+08 | 85336000 | 73586000 | 89086000 | 68676000 |
| F10 | na | 30089000 | 63629000 | 27652000 | 29556000 | 28517000 | 38741000 | 30437000 | 43659000 | 0 |
| EXTL2 | na | 2.81E+09 | 74144000 | 31076000 | 9.32E+08 | 77306000 | 1.81E+08 | 5E+08 | 9078700 | 4.46E+08 |
| ERN1 | na | 0 | 0 | 0 | 0 | 0 | 0 | 0 | 0 | 2.97E+08 |
| EPX | na | 30870000 | 0 | 21652000 | 9748600 | 46124000 | 21018000 | 19063000 | 22498000 | 97066000 |
| EPB42 | na | 0 | 0 | 0 | 42546000 | 31356000 | 50839000 | 0 | 23477000 | 0 |
| ENTPD8 | na | 0 | 0 | 7293000 | 0 | 0 | 0 | 0 | 0 | 0 |
| ENO1 | na | 71827000 | 7755700 | 18699000 | 19868000 | 43435000 | 19188000 | 10878000 | 16919000 | 0 |
| EMILIN1 | na | 0 | 0 | 0 | 0 | 0 | 4484800 | 0 | 11395000 | 0 |
| ELANE | na | 0 | 0 | 0 | 0 | 0 | 65634000 | 27611000 | 0 | 0 |
| EHD3 | na | 2.75E+08 | 0 | 75252000 | 37601000 | 49650000 | 47449000 | 18355000 | 13893000 | 0 |
| EFEMP1 | na | 0 | 7770100 | 11586000 | 18280000 | 14054000 | 0 | 0 | 11440000 | 19758000 |
| ECM1 | na | 3.65E+08 | 8.06E+08 | 3.66E+08 | 3.77E+08 | 5.27E+08 | 3.28E+08 | 5.74E+08 | 3.15E+08 | 1.13E+08 |
| EARS2 | na | 14927000 | 14921000 | 0 | 4823300 | 10918000 | 16700000 | 0 | 13828000 | 32572000 |
| DSTN | na | 60187000 | 0 | 7628100 | 6146500 | 5656600 | 0 | 0 | 0 | 0 |
| DSP | na | 9132200 | 0 | 23490000 | 20259000 | 34827000 | 0 | 0 | 0 | 19210000 |
| DSG1 | na | 0 | 0 | 24283000 | 0 | 0 | 0 | 0 | 0 | 0 |
| DSC1 | na | 0 | 1.01E+08 | 0 | 84057000 | 41412000 | 67646000 | 44136000 | 39456000 | 0 |
| DPP4 | na | 7876400 | 0 | 2.75E+08 | 17492000 | 7713700 | 15315000 | 0 | 12731000 | 7690400 |
| DLST | na | 40469000 | 0 | 32492000 | 6086800 | 26918000 | 15199000 | 7980600 | 0 | 14149000 |
| DERA | na | 11101000 | 0 | 4962700 | 7070600 | 14641000 | 0 | 0 | 0 | 0 |
| DEFA3 | na | 0 | 0 | 43499000 | 63005000 | 1.97E+08 | 1.13E+08 | 85619000 | 60034000 | 1.37E+08 |
| DCD | na | 1.05E+08 | 0 | 44761000 | 0 | 51164000 | 37195000 | 0 | 0 | 56677000 |
| DBH | na | 8484100 | 0 | 0 | 0 | 7990500 | 12729000 | 18637000 | 18746000 | 9853200 |
| CYFIP1 | na | 1.72E+08 | 4927600 | 44095000 | 79130000 | 77468000 | 88922000 | 26443000 | 21135000 | 5357400 |
| CTSG | na | 5522200 | 2801200 | 51622000 | 43599000 | 1.58E+08 | 1.78E+08 | 29706000 | 83070000 | 1.02E+08 |
| CRTAC1 | na | 0 | 14208000 | 0 | 0 | 0 | 0 | 0 | 0 | 0 |
| CRP | na | 0 | 3227200 | 7043300 | 13928000 | 1.22E+08 | 3317600 | 7983000 | 9.28E+08 | 9772200 |
| CPNE3 | na | 14626000 | 0 | 0 | 18616000 | 27634000 | 19762000 | 13034000 | 13473000 | 25558000 |
| CPNE1 | na | 32132000 | 7187100 | 17828000 | 21641000 | 31121000 | 65689000 | 10009000 | 23888000 | 6.83E+08 |
| CPN2 | na | 1.71E+08 | 4.26E+08 | 1.73E+08 | 3.51E+08 | 2.75E+08 | 3.39E+08 | 3.87E+08 | 5.53E+08 | 96197000 |
| CPN1 | na | 0 | 1.42E+08 | 29244000 | 1.12E+08 | 69433000 | 1.02E+08 | 1.51E+08 | 1.95E+08 | 16518000 |
| CPB2 | na | 0 | 3.69E+08 | 24373000 | 3.32E+08 | 1.21E+08 | 1.63E+08 | 1.72E+08 | 2.42E+08 | 24753000 |
| CP | na | 1.33E+09 | 2.21E+10 | 2.35E+09 | 1.01E+10 | 8.83E+09 | 1.02E+10 | 9.86E+09 | 1.32E+10 | 4.45E+09 |
| COTL1 | na | 17287000 | 0 | 5663300 | 2841500 | 4727600 | 3661600 | 2279300 | 2588600 | 0 |
| CORO1A | na | 43098000 | 0 | 17347000 | 15744000 | 0 | 0 | 31729000 | 0 | 0 |
| COLEC11 | na | 48607000 | 39019000 | 29738000 | 55966000 | 55012000 | 64029000 | 33572000 | 52515000 | 36175000 |
| COLEC10 | na | 17042000 | 0 | 0 | 14779000 | 15843000 | 19048000 | 0 | 16057000 | 0 |
| COL6A3 | na | 2.21E+08 | 23475000 | 2.04E+08 | 1.12E+08 | 80623000 | 71237000 | 2.06E+08 | 48088000 | 81384000 |
| COL6A2 | na | 46421000 | 0 | 33199000 | 16902000 | 15895000 | 11926000 | 24304000 | 0 | 19463000 |
| COL6A1 | na | 58041000 | 0 | 54296000 | 54894000 | 32380000 | 31256000 | 67376000 | 37361000 | 76459000 |
| COL1A2 | na | 0 | 0 | 77790000 | 0 | 0 | 0 | 0 | 0 | 0 |
| CNDP1 | na | 0 | 1.53E+08 | 0 | 0 | 12023000 | 43752000 | 9976500 | 25469000 | 0 |
| CLU | na | 4.54E+09 | 6.44E+09 | 5.37E+09 | 5.23E+09 | 6.14E+09 | 6.24E+09 | 4.65E+09 | 6.16E+09 | 1.46E+10 |
| CLTC | na | 0 | 0 | 0 | 0 | 1.99E+08 | 2.05E+08 | 19887000 | 27713000 | 6258000 |
| CLIC1 | na | 56676000 | 0 | 15035000 | 15098000 | 10764000 | 14388000 | 8599600 | 14488000 | 11214000 |
| CLEC3B | na | 0 | 1.82E+08 | 27335000 | 74161000 | 57835000 | 53910000 | 52448000 | 49174000 | 0 |
| CLDN5 | na | 11802000 | 0 | 6869600 | 0 | 4126400 | 0 | 7017500 | 0 | 0 |
| CLCA1 | na | 0 | 0 | 0 | 0 | 31659000 | 0 | 0 | 0 | 0 |
| CHMP4A | na | 1.58E+08 | 69391000 | 1.78E+08 | 53100000 | 2.37E+08 | 1.08E+08 | 1.22E+08 | 2.76E+08 | 1.69E+08 |
| CFP | na | 3E+09 | 1.16E+09 | 5.78E+09 | 6.86E+08 | 9.97E+08 | 2.28E+09 | 3.74E+08 | 6.78E+08 | 1.22E+09 |
| CFL1 | na | 2.79E+09 | 1.55E+08 | 1.08E+09 | 6.6E+08 | 6.91E+08 | 6.04E+08 | 3.8E+08 | 2.31E+08 | 2.39E+08 |
| CFI | na | 4.46E+08 | 1.55E+09 | 6.77E+08 | 1.29E+09 | 1.14E+09 | 9.22E+08 | 1.03E+09 | 1.15E+09 | 5.73E+08 |
| CFHR5 | na | 65258000 | 0 | 19057000 | 30687000 | 26133000 | 30272000 | 19640000 | 37213000 | 24845000 |
| CFHR4 | na | 0 | 0 | 0 | 10327000 | 28745000 | 0 | 9926000 | 15524000 | 9092100 |
| CFHR2 | na | 0 | 24011000 | 0 | 19572000 | 22501000 | 0 | 30475000 | 0 | 0 |
| CFHR1 | na | 1.12E+08 | 6.7E+08 | 2.91E+08 | 4.62E+08 | 4.61E+08 | 2.55E+08 | 3.88E+08 | 8.36E+08 | 2.93E+08 |
| CFH | na | 2.67E+09 | 2.33E+10 | 3.71E+09 | 1.28E+10 | 8.5E+09 | 9.39E+09 | 8.71E+09 | 1.21E+10 | 3.61E+09 |
| CFD | na | 0 | 20731000 | 0 | 4964400 | 3432300 | 0 | 0 | 4281500 | 0 |
| CFB | na | 7.68E+08 | 8.18E+09 | 1.03E+09 | 3.25E+09 | 2.62E+09 | 3.48E+09 | 2.52E+09 | 4.65E+09 | 5.87E+08 |
| CETP | na | 1.01E+08 | 29361000 | 1.12E+08 | 21899000 | 31670000 | 15329000 | 19013000 | 27820000 | 77123000 |
| CEMIP | na | 12833000 | 0 | 37715000 | 30247000 | 12160000 | 8979000 | 27179000 | 10255000 | 26635000 |
| CDC5L | na | 5.6E+09 | 6.95E+08 | 3.08E+09 | 2.24E+09 | 2.15E+09 | 1.65E+09 | 2.32E+09 | 1.35E+09 | 4.53E+09 |
| CD9 | na | 1.06E+09 | 2.49E+08 | 1.96E+09 | 1.87E+09 | 1.96E+09 | 2.23E+09 | 1.46E+09 | 1.15E+09 | 9.73E+08 |
| CD82 | na | 36299000 | 0 | 35586000 | 52886000 | 43548000 | 26186000 | 0 | 4365500 | 26736000 |
| CD81 | na | 37134000 | 0 | 37997000 | 21782000 | 20855000 | 17853000 | 12656000 | 10541000 | 17814000 |
| CD63 | na | 1.88E+08 | 0 | 2.21E+08 | 0 | 2.44E+08 | 0 | 1.99E+08 | 0 | 1.28E+08 |
| CD5L | na | 2.32E+11 | 2.34E+10 | 1.41E+11 | 7.48E+10 | 4.94E+10 | 6.09E+10 | 1.33E+11 | 6.52E+10 | 2.23E+11 |
| CD47 | na | 48277000 | 5906400 | 24971000 | 35287000 | 34041000 | 34812000 | 8046500 | 19179000 | 19596000 |
| CD36 | na | 6.17E+08 | 66287000 | 3.78E+08 | 3.65E+08 | 5.31E+08 | 2.35E+08 | 2.22E+08 | 2.71E+08 | 4.37E+08 |
| CD226 | na | 15940000 | 0 | 0 | 5386800 | 3125700 | 4840000 | 0 | 0 | 0 |
| CD151 | na | 1.12E+08 | 0 | 1.21E+08 | 0 | 0 | 0 | 86319000 | 61155000 | 40350000 |
| CD14 | na | 2628000 | 21620000 | 6784700 | 3008000 | 6157700 | 4352500 | 0 | 6281600 | 4974900 |
| CCT4 | na | 0 | 0 | 1186000 | 5675300 | 2435200 | 9590500 | 0 | 818770 | 1051300 |
| CCDC73 | na | 0 | 23495000 | 0 | 0 | 0 | 14126000 | 0 | 0 | 75673000 |
| CAVIN2 | na | 79801000 | 0 | 30610000 | 98145000 | 60672000 | 53186000 | 39630000 | 9258800 | 19722000 |
| CAT | na | 24670000 | 24289000 | 45911000 | 92065000 | 79417000 | 79371000 | 28100000 | 1.89E+08 | 32038000 |
| CAPZA1 | na | 51690000 | 0 | 15126000 | 11963000 | 13614000 | 11222000 | 0 | 6264300 | 6158400 |
| CAP1 | na | 2.57E+08 | 0 | 16328000 | 24255000 | 38923000 | 69432000 | 9005600 | 12069000 | 10938000 |
| CANX | na | 0 | 0 | 0 | 0 | 9777100 | 0 | 0 | 0 | 0 |
| CAMP | na | 26123000 | 0 | 24041000 | 30734000 | 52515000 | 38473000 | 15242000 | 7716700 | 33512000 |
| CAMK2A | na | 0 | 0 | 7852200 | 0 | 0 | 0 | 0 | 0 | 0 |
| CALM3 | na | 1.35E+08 | 15345000 | 59453000 | 54379000 | 63185000 | 44114000 | 23532000 | 52225000 | 33308000 |
| CA2 | na | 0 | 0 | 0 | 21900000 | 22805000 | 0 | 0 | 29289000 | 0 |
| CA1 | na | 37879000 | 16323000 | 51834000 | 2.85E+08 | 1.33E+08 | 1.52E+08 | 13766000 | 2.06E+08 | 17352000 |
| C9 | na | 2.74E+09 | 2.13E+09 | 2.81E+09 | 2.82E+09 | 3.56E+09 | 4.17E+09 | 1.88E+09 | 3.24E+09 | 1.48E+10 |
| C8G | na | 3.99E+08 | 1.3E+09 | 3.26E+08 | 1.01E+09 | 8.49E+08 | 1.43E+09 | 1.16E+09 | 1.11E+09 | 2.19E+09 |
| C8B | na | 1.1E+09 | 2.7E+09 | 1.29E+09 | 2.65E+09 | 2.45E+09 | 3.48E+09 | 3.49E+09 | 2.42E+09 | 5.63E+09 |
| C8A | na | 7.93E+08 | 1.85E+09 | 1E+09 | 1.7E+09 | 1.64E+09 | 2.21E+09 | 2.07E+09 | 2E+09 | 4.52E+09 |
| C7 | na | 7.29E+08 | 2.14E+09 | 5.57E+08 | 1.29E+09 | 1.03E+09 | 1.78E+09 | 1.27E+09 | 1.12E+09 | 4.37E+09 |
| C6 | na | 6.93E+08 | 1.88E+09 | 4.83E+08 | 9.22E+08 | 9.47E+08 | 1.39E+09 | 7.06E+08 | 9.39E+08 | 4.47E+09 |
| C5 | na | 3.92E+09 | 1.1E+10 | 3.42E+09 | 1.3E+10 | 7.42E+09 | 1.13E+10 | 9.53E+09 | 1.17E+10 | 1.8E+10 |
| C4BPB | na | 4.85E+09 | 1.14E+09 | 9.27E+09 | 3.34E+09 | 3.15E+09 | 2.82E+09 | 5.19E+09 | 4.46E+09 | 7.59E+09 |
| C4BPA | na | 7.71E+10 | 2.56E+10 | 1.34E+11 | 6.55E+10 | 4.04E+10 | 4.96E+10 | 8.8E+10 | 7.32E+10 | 1.10E+11 |
| C4B | na | 1.04E+11 | 5.11E+10 | 1.43E+11 | 4.61E+10 | 4.98E+10 | 6.87E+10 | 6.61E+10 | 9.3E+10 | 1.33E+11 |
| C4A | na | 4.37E+09 | 1.65E+09 | 3.54E+09 | 1.36E+09 | 1.02E+09 | 1.21E+09 | 1.28E+09 | 2.12E+09 | 2.22E+09 |
| C3 | na | 1.10E+11 | 1.78E+11 | 9.23E+10 | 1.47E+11 | 8.44E+10 | 1.32E+11 | 1.09E+11 | 1.58E+11 | 7.41E+10 |
| C2 | na | 5676700 | 5.28E+08 | 8646600 | 89047000 | 67648000 | 2.28E+08 | 1.5E+08 | 2.31E+08 | 5276300 |
| C1S | na | 1.02E+10 | 8.65E+09 | 1.61E+10 | 3.32E+10 | 3.22E+10 | 2.5E+10 | 3.28E+10 | 2.22E+10 | 1.43E+10 |
| C1RL | na | 0 | 36731000 | 0 | 0 | 0 | 0 | 9915600 | 10440000 | 0 |
| C1R | na | 1.27E+10 | 1.24E+10 | 2.25E+10 | 4.66E+10 | 3.86E+10 | 3.15E+10 | 4.99E+10 | 2.96E+10 | 1.81E+10 |
| C1QTNF3 | na | 1.04E+08 | 0 | 1.37E+08 | 0 | 0 | 0 | 0 | 0 | 1.83E+08 |
| C1QC | na | 1.55E+10 | 8.93E+09 | 1.31E+10 | 3.02E+10 | 2.91E+10 | 2.4E+10 | 3.88E+10 | 2.04E+10 | 2.1E+10 |
| C1QB | na | 1.44E+10 | 9.47E+09 | 1.84E+10 | 3.24E+10 | 2.37E+10 | 2.65E+10 | 3.15E+10 | 2.08E+10 | 1.93E+10 |
| C1QA | na | 1.26E+10 | 6.32E+09 | 1.35E+10 | 2.81E+10 | 2.32E+10 | 2.22E+10 | 2.98E+10 | 1.64E+10 | 1.71E+10 |
| C16orf54 | na | 0 | 0 | 9397900 | 0 | 0 | 4254600 | 0 | 0 | 8968500 |
| C16orf46 | na | 44082000 | 54472000 | 50390000 | 0 | 0 | 59078000 | 53754000 | 27716000 | 74378000 |
| BTD | na | 0 | 37113000 | 0 | 3771600 | 0 | 0 | 0 | 9049200 | 0 |
| BPI | na | 92012000 | 18291000 | 43036000 | 32612000 | 1.16E+08 | 80025000 | 0 | 22485000 | 32192000 |
| BLVRB | na | 0 | 0 | 0 | 0 | 0 | 0 | 0 | 3697600 | 0 |
| BLMH | na | 5749100 | 0 | 5694000 | 5437500 | 29195000 | 3408700 | 4254800 | 6079600 | 0 |
| BCHE | na | 38632000 | 2.47E+08 | 71863000 | 1.33E+08 | 1.25E+08 | 1.21E+08 | 3.72E+08 | 1.49E+08 | 40500000 |
| BANF1 | na | 0 | 0 | 0 | 0 | 25759000 | 14638000 | 0 | 18097000 | 24208000 |
| B2M | na | 64937000 | 1.1E+08 | 97139000 | 76466000 | 1.1E+08 | 86630000 | 77108000 | 52866000 | 64315000 |
| AZU1 | na | 0 | 0 | 0 | 0 | 1.32E+08 | 1.02E+08 | 0 | 66150000 | 0 |
| AZGP1 | na | 58101000 | 4.31E+09 | 2.9E+08 | 5.97E+08 | 8.24E+08 | 6.69E+08 | 8.9E+08 | 7.48E+08 | 1.08E+08 |
| ATRN | na | 0 | 3.82E+08 | 51873000 | 1.82E+08 | 96922000 | 89046000 | 1.38E+08 | 1.21E+08 | 28149000 |
| ATP6V0A1 | na | 0 | 0 | 0 | 10855000 | 11964000 | 0 | 0 | 11459000 | 18214000 |
| ATP2C1 | na | 0 | 0 | 0 | 0 | 0 | 0 | 0 | 0 | 0 |
| ATP2A3 | na | 0 | 0 | 0 | 8903800 | 8122900 | 0 | 0 | 0 | 0 |
| ARPC3 | na | 29430000 | 0 | 0 | 13041000 | 28932000 | 13380000 | 0 | 0 | 0 |
| ARPC2 | na | 86773000 | 0 | 0 | 19972000 | 27022000 | 27918000 | 13519000 | 16356000 | 0 |
| ARPC1B | na | 22649000 | 0 | 8335200 | 7507300 | 5287400 | 8421400 | 9800400 | 7352900 | 0 |
| ARL6IP5 | na | 0 | 0 | 0 | 0 | 17895000 | 0 | 0 | 5903500 | 5943700 |
| ARF1 | na | 0 | 0 | 0 | 10579000 | 0 | 0 | 0 | 0 | 0 |
| AQP1 | na | 0 | 0 | 0 | 8829000 | 9199900 | 0 | 0 | 0 | 0 |
| APOM | na | 1.59E+08 | 2.7E+08 | 1.01E+08 | 91182000 | 67495000 | 1.12E+08 | 1.39E+08 | 1.02E+08 | 1.05E+08 |
| APOL1 | na | 1.37E+10 | 1.79E+09 | 1.03E+10 | 1E+10 | 4.65E+09 | 3.43E+09 | 1.28E+10 | 5.51E+09 | 2.05E+10 |
| APOH | na | 3.36E+08 | 6.49E+09 | 7.93E+08 | 1.73E+09 | 1.7E+09 | 1.75E+09 | 2.01E+09 | 1.93E+09 | 4.3E+08 |
| APOF | na | 0 | 49166000 | 80166000 | 0 | 11508000 | 13464000 | 2.78E+08 | 0 | 0 |
| APOE | na | 5.89E+09 | 5.96E+09 | 7.44E+09 | 3.88E+09 | 8.65E+09 | 3.34E+09 | 3.32E+09 | 4.94E+09 | 9.36E+09 |
| APOD | na | 5.4E+08 | 1.22E+09 | 6.15E+08 | 6.54E+08 | 1.19E+09 | 8.53E+08 | 6.53E+08 | 9.18E+08 | 4.31E+08 |
| APOC4 | na | 35687000 | 70141000 | 65804000 | 32214000 | 34975000 | 16788000 | 38989000 | 74362000 | 47740000 |
| APOC3 | na | 9.47E+08 | 4.82E+09 | 2.21E+09 | 2.2E+09 | 2.43E+09 | 1.67E+09 | 2.37E+09 | 2.38E+09 | 1.19E+09 |
| APOC2 | na | 1.59E+08 | 5.81E+08 | 4.63E+08 | 3.08E+08 | 5.94E+08 | 1.56E+08 | 3.93E+08 | 5.53E+08 | 2.6E+08 |
| APOC1 | na | 6.87E+08 | 3.42E+09 | 8.75E+08 | 8.14E+08 | 1.18E+09 | 5.55E+08 | 7.58E+08 | 7.92E+08 | 6.88E+08 |
| APOB | na | 2.77E+10 | 4.41E+10 | 5.37E+10 | 3.32E+10 | 3.92E+10 | 2.84E+10 | 2.29E+10 | 3.46E+10 | 2.59E+10 |
| APOA5 | na | 0 | 0 | 9924500 | 0 | 0 | 6586300 | 0 | 0 | 12456000 |
| APOA4 | na | 2.68E+09 | 7.58E+09 | 3.12E+09 | 4.1E+09 | 3.96E+09 | 5.62E+09 | 4.53E+09 | 2.71E+09 | 4.45E+09 |
| APOA2 | na | 2.8E+09 | 1.64E+10 | 5.99E+09 | 5.71E+09 | 5.61E+09 | 6.99E+09 | 8.06E+09 | 5.57E+09 | 4.16E+09 |
| APOA1 | na | 7.1E+10 | 1.59E+11 | 7.18E+10 | 5.76E+10 | 4.51E+10 | 5.49E+10 | 7.07E+10 | 5.02E+10 | 7.69E+10 |
| APMAP | na | 11588000 | 0 | 5891100 | 13941000 | 16645000 | 8937700 | 13941000 | 11270000 | 0 |
| APCS | na | 1.24E+09 | 2.91E+09 | 5.68E+08 | 3.85E+09 | 2.55E+09 | 3.33E+09 | 2.76E+09 | 5.03E+09 | 1.41E+09 |
| ANXA7 | na | 4.36E+08 | 17027000 | 3.25E+08 | 8.11E+08 | 1.86E+09 | 5.53E+08 | 3.72E+08 | 7.36E+08 | 4.62E+08 |
| ANXA6 | na | 0 | 0 | 0 | 19490000 | 23061000 | 17471000 | 0 | 15781000 | 37950000 |
| ANXA5 | na | 77450000 | 0 | 68579000 | 1.23E+08 | 1.89E+08 | 1.82E+08 | 71384000 | 82125000 | 1.17E+08 |
| ANXA4 | na | 24167000 | 11197000 | 52016000 | 49233000 | 3.19E+08 | 1.39E+08 | 1.76E+08 | 72933000 | 1.11E+08 |
| ANXA3 | na | 1.45E+08 | 14177000 | 98142000 | 1.14E+08 | 2.96E+08 | 2.08E+08 | 66040000 | 1.27E+08 | 1.46E+08 |
| ANXA2 | na | 0 | 0 | 3813900 | 0 | 4343200 | 0 | 0 | 2944600 | 0 |
| ANXA11 | na | 3.44E+08 | 33270000 | 4.05E+08 | 5E+08 | 1.45E+09 | 8.49E+08 | 3.39E+08 | 5.62E+08 | 5.68E+08 |
| ANXA1 | na | 0 | 0 | 0 | 0 | 18468000 | 8013600 | 0 | 2323400 | 39016000 |
| ANPEP | na | 1.23E+08 | 10806000 | 6.44E+09 | 4.31E+08 | 1.67E+08 | 1.38E+08 | 73223000 | 1.5E+08 | 4.51E+08 |
| ANK1 | na | 0 | 0 | 0 | 6611900 | 13986000 | 13015000 | 0 | 6167900 | 0 |
| ANGPT1 | na | 9.3E+08 | 78498000 | 1.26E+08 | 1.96E+08 | 1.43E+08 | 43373000 | 1.88E+08 | 1.91E+08 | 1.74E+08 |
| AMBP | na | 4.25E+08 | 2.38E+09 | 5.81E+08 | 7.03E+08 | 9.86E+08 | 1.25E+09 | 1E+09 | 1.63E+09 | 4.44E+08 |
| ALOX5AP | na | 0 | 0 | 0 | 7874400 | 26553000 | 13549000 | 0 | 8970400 | 14916000 |
| ALDH16A1 | na | 18336000 | 0 | 13125000 | 11352000 | 9257100 | 12537000 | 0 | 0 | 9946700 |
| ALAD | na | 3814700 | 2095100 | 2.22E+08 | 6495400 | 0 | 9469800 | 0 | 0 | 0 |
| AHSG | na | 4.39E+08 | 1.23E+10 | 1.7E+09 | 1.33E+09 | 1.72E+09 | 2.06E+09 | 1.43E+09 | 1.51E+09 | 5.91E+08 |
| AHNAK2 | na | 0 | 0 | 0 | 0 | 0 | 0 | 0 | 0 | 1.08E+08 |
| AGT | na | 1.07E+09 | 2.65E+09 | 3.38E+08 | 7.65E+08 | 5.09E+08 | 6.85E+08 | 6.38E+08 | 1.02E+09 | 3.14E+08 |
| AFM | na | 36993000 | 2.55E+09 | 1.68E+08 | 3.4E+08 | 4.79E+08 | 5.55E+08 | 6.28E+08 | 4.95E+08 | 38548000 |
| ADIPOQ | na | 2.6E+09 | 1.23E+08 | 2.69E+08 | 1.27E+08 | 5.81E+08 | 3.59E+08 | 6.81E+08 | 2.15E+08 | 1.83E+08 |
| ADAM10 | na | 6.71E+08 | 99675000 | 7.94E+08 | 5.45E+08 | 1.11E+09 | 6.62E+08 | 5.06E+08 | 2.9E+08 | 3.76E+08 |
| ACTR3 | na | 76568000 | 0 | 10275000 | 16171000 | 20810000 | 21809000 | 0 | 10497000 | 3969700 |
| ACTR2 | na | 77615000 | 0 | 13367000 | 17603000 | 23512000 | 17574000 | 20286000 | 0 | 0 |
| ACTN1 | na | 8.62E+08 | 40610000 | 2.21E+08 | 2.6E+08 | 3.13E+08 | 3.3E+08 | 68513000 | 1.41E+08 | 47185000 |
| ACTG1 | na | 0 | 0 | 16091000 | 0 | 0 | 0 | 0 | 0 | 0 |
| ACTB | na | 2.7E+10 | 1.09E+09 | 7.97E+09 | 8.35E+09 | 8.38E+09 | 9.24E+09 | 4.6E+09 | 4.64E+09 | 2.84E+09 |
| ACTA1 | na | 2.55E+09 | 74897000 | 8.95E+08 | 6.8E+08 | 5.84E+08 | 6.2E+08 | 3.71E+08 | 3.87E+08 | 2.21E+08 |
| ABCB11 | na | 17355000 | 0 | 13748000 | 7139300 | 0 | 0 | 0 | 0 | 0 |
| A2M | na | 1.04E+12 | 5.02E+11 | 7.58E+11 | 9.92E+11 | 6.61E+11 | 6.82E+11 | 1.37E+12 | 7.44E+11 | 1.22E+12 |
| A1BG | na | 1.74E+08 | 8.92E+09 | 5.66E+08 | 9.31E+08 | 1.04E+09 | 1.39E+09 | 1.21E+09 | 1.71E+09 | 2.15E+08 |
